# Complex macrocycle exploration: parallel, heuristic, and constraint-based conformer generation using ForceGen

**DOI:** 10.1007/s10822-019-00203-1

**Published:** 2019-05-03

**Authors:** Ajay N. Jain, Ann E. Cleves, Qi Gao, Xiao Wang, Yizhou Liu, Edward C. Sherer, Mikhail Y. Reibarkh

**Affiliations:** 1 Department of Bioengineering and Therapeutic Sciences, University of California, San Francisco, USA; 2 Applied Science, BioPharmics LLC, Santa Rosa, CA USA; 3 Process Research and Development, Merck & Co., Inc., Kenilworth, NJ USA; 4 Present Address: Analytical Research and Development, Pfizer Inc., Groton, CT USA; 5 Modeling and Informatics, Merck & Co., Inc., Kenilworth, NJ USA

**Keywords:** ForceGen, Conformer generation, Macrocycle, Multi-core, Surflex, NMR, RDC

## Abstract

ForceGen is a template-free, non-stochastic approach for 2D to 3D structure generation and conformational elaboration for small molecules, including both non-macrocycles and macrocycles. For conformational search of non-macrocycles, ForceGen is both faster and more accurate than the best of all tested methods on a very large, independently curated benchmark of 2859 PDB ligands. In this study, the primary results are on macrocycles, including results for 431 unique examples from four separate benchmarks. These include complex peptide and peptide-like cases that can form networks of internal hydrogen bonds. By making use of new physical movements (“flips” of near-linear sub-cycles and explicit formation of hydrogen bonds), ForceGen exhibited statistically significantly better performance for overall RMS deviation from experimental coordinates than all other approaches. The algorithmic approach offers natural parallelization across multiple computing-cores. On a modest multi-core workstation, for all but the most complex macrocycles, median wall-clock times were generally under a minute in fast search mode and under 2 min using thorough search. On the most complex cases (roughly cyclic decapeptides and larger) explicit exploration of likely hydrogen bonding networks yielded marked improvements, but with calculation times increasing to several minutes and in some cases to roughly an hour for fast search. In complex cases, utilization of NMR data to constrain conformational search produces accurate conformational ensembles representative of solution state macrocycle behavior. On macrocycles of typical complexity (up to 21 rotatable macrocyclic and exocyclic bonds), design-focused macrocycle optimization can be practically supported by computational chemistry at interactive time-scales, with conformational ensemble accuracy equaling what is seen with non-macrocyclic ligands. For more complex macrocycles, inclusion of sparse biophysical data is a helpful adjunct to computation.

## Introduction

ForceGen is a method for 3D structure generation and conformational elaboration that does not rely on distance geometry [1–6], precalculated molecular templates [7, 8], or stochastic sampling [9–11]. It is driven by coupling intuitive physical molecular movement with the internal conformational energy computed from a molecular mechanics force field (MMFF94sf [12–18]). For full details on the ForceGen method, please refer to the original publication [18], which included comparisons to other methods on both non-macrocycles and macrocycles, though comparisons were limited in scope and could not include more recent alternative method performance data. The primary changes within the ForceGen methodology reported here have been in the area of macrocycles, so detailed discussion and comparative analysis will be presented on large macrocycle-focused benchmarks. Additional speed optimization has been done, in particular making use of parallel calculations on multi-core workstations.

**Table 1:** Summary of molecular datasets and their relative complexity

Set name	Description	N	N heavy atoms	Rot. bonds	N macrocycles	Macro. size
Platinum [19]	Large PDB benchmark	2859	24.1 ± 8.2	5.5 ± 3.2	29	14.7 ± 6.1
Chen/Foloppe [20]	Diverse macrocycles	30	39.6 ± 15.6	6.2 ± 5.1	30	18.2 ± 8.3
ForceGen [18]	Diverse macrocycles	182	40.0 ± 11.1	6.7 ± 4.5	182	16.7 ± 4.3
Shelley [11]	Diverse macrocycles	150	49.5 ± 18.7	7.8 ± 6.1	150	20.2 ± 6.9
Prime-MCS [21]	Diverse macrocycles	208	42.3 ± 21.8	6.0 ± 6.9	208	19.3 ± 7.1

ForceGen uses exactly the same algorithmic machinery to search non-macrocycles and macrocycles. Using the large Platinum Diverse Dataset (99% non-macrocycles) from the recent paper of Kirchmair’s group [19], a brief summary of ForceGen’s performance will be made to facilitate comparison with recent versions of other widely used methods. However, the primary focus in this study is on macrocyclic ligands. The original ForceGen report showed comparative performance using 30 macrocyclic ligands from the widely used Chen and Foloppe benchmark [20], but more substantial analysis was presented on a set of 182 macrocycles curated from the PDB, which will be used here to compare current and prior ForceGen performance. In this report, we add a 150 macrocycle data set curated from the CSD and PDB in a detailed study of the MacroModel Large-Scale Low-Mode approach [11] and a recently published 208 molecule macrocycle set reported with the introduction of the Prime-MCS approach [21]. The latter study included comparative performance for a number of widely used methods. The overall complexity of the four data sets is summarized in Table 1. In total, the data include 431 unique macrocyclic ligands, forming the largest such set analyzed in a single study.

**Fig. 1:**
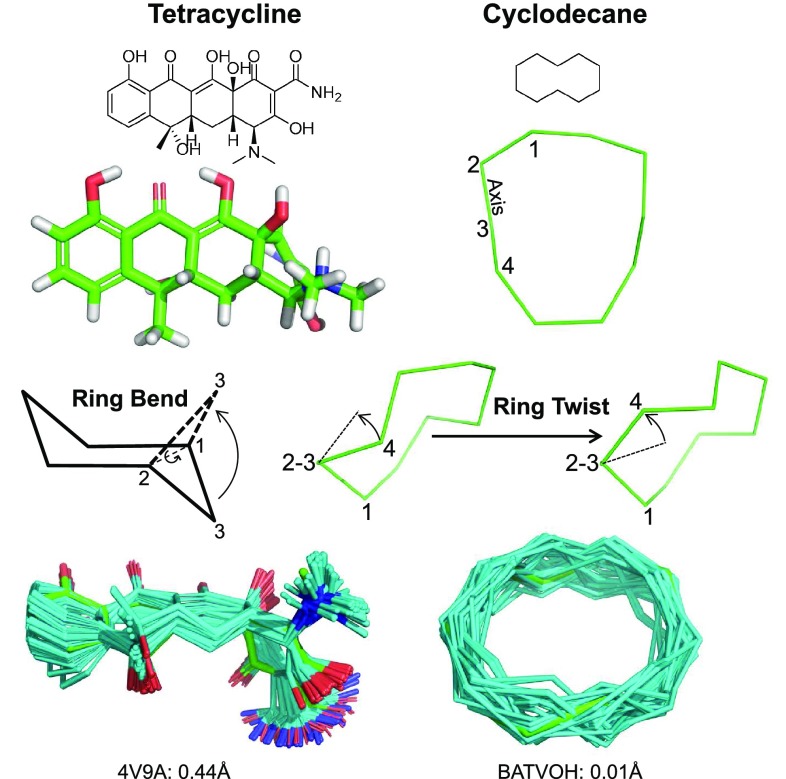
Ring bending for elaboration of ring system flexibility: initial 3D structure generation produced a reasonable conformer for tetracycline (upper left); ring bends are identified among atoms of a ring system according to rules, with an example for cyclohexane shown (middle left); iterative application of the bends identifies new ring conformations effectively (bottom left). Ring twisting for macrocyclic search: the initial structure of cyclodecane (upper right) is shown with four atoms marked; those atoms seen through the 2–3 axis (middle right) are pushed through a twisting motion where atom 4 is forced around the axis; iterative application of this strategy results in an effective enumeration of ring conformers for cyclodecane

ForceGen’s approach to conformer generation is driven by the force field (a variant of MMFF94s). Figure 1 (left side) illustrates the search method for ring systems composed of multiple small flexible rings, exemplified by tetracycline. The central concept is the “bend.” Such bending replicates the intuitive physical manipulation of a plastic organic chemistry model of cyclohexane to produce chair, twist-boat, and boat conformations. Following identification of ring systems, ring atom pairs are identified across which to make a bend. Bends are repeatedly applied to an evolving set of distinct low energy ring conformations, with each bend requiring a direct movement of atoms followed by a careful force field minimization procedure that avoids reversion. Additional details of the bending process are given in the Methods Section. For tetracycline, ring system identification yields the fused set of four six-membered rings (each with different saturation patterns). The repeated process of bending the ring system yields an ensemble of 117 low-energy conformers. The procedure is general, not requiring any precomputation of large numbers of specific ring templates, and its pure physical manipulation is effective on diverse ring systems.

For macrocyclic systems, the components that are composed of small rings are elaborated using the bending approach just described. For ring systems of size 9 or larger, ForceGen makes use of an additional physical manipulation: a “twist” that is applied to force rotation around the bonds within macrocycles. Figure 1 (right side) illustrates this using cyclodecane as an example. The approach is very similar to that described for application of ring bends, with force being applied to push a macrocycle atom around a torsional axis (see the Methods Section for additional details). This simple procedure produces 245 distinct conformations for cyclodecane in about 10 s on a four-core workstation.

**Fig. 2:**
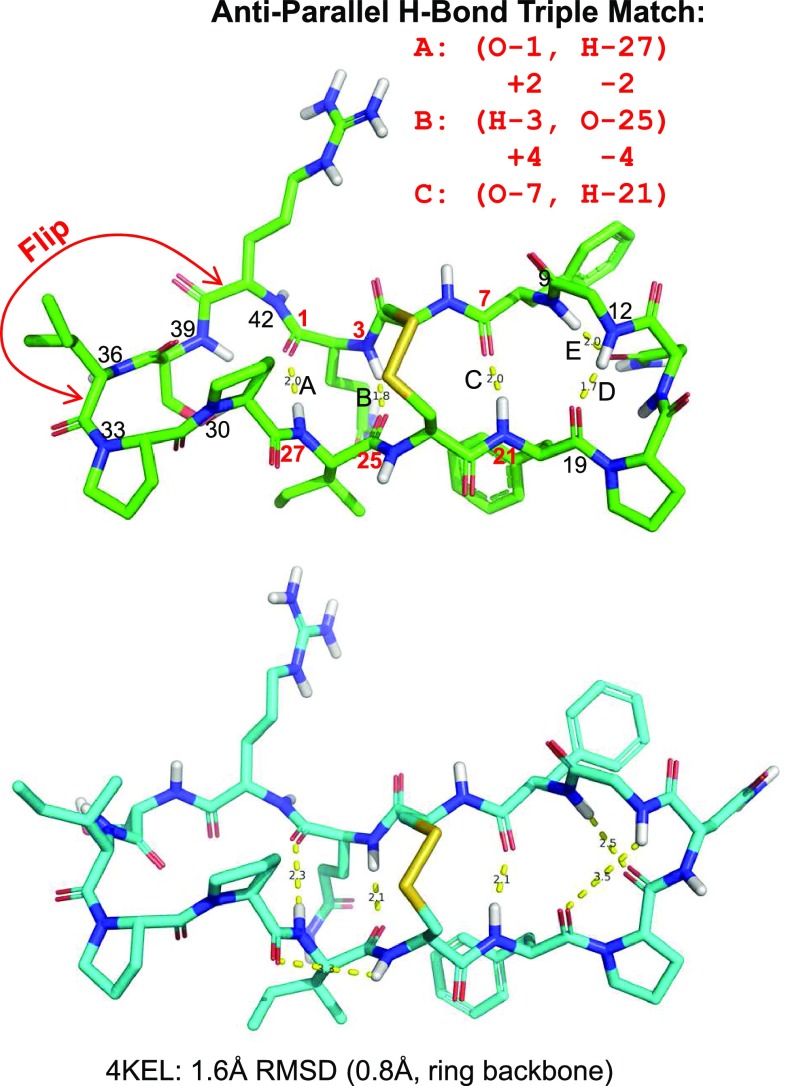
New physical movements include flips of stretches of macrocycle sub-rings and coordinated formation of multiple hydrogen bonds (top); best matching conformer from ForceGen thorough search (bottom, shown in cyan)

In this work, two additional physical movements are introduced (see Fig. 2), which aid in macrocyclic conformer elucidation. The first is the “flip” which identifies nearly co-linear macrocyclic ring bond pairs that are rotatable (marked by red arrows). The degree to which the bonds are consistent with a sensible flipping motion is assessed by considering the bond vectors themselves and the vectors between mid-points of the pair of bonds. If the two bonds point in similar directions and they also point in the same direction as the vector between their mid-points, then a flip is made. In Fig. 2, the indicated flip will cause a coordinated movement of two residues (an isoleucine and a serine).

The second physical movement is the explicit formation of cross-macrocycle hydrogen bonds. For large peptide macrocycles, such interactions are often critical features of stable low-energy conformations. This requires the identification of likely hydrogen bonding pairs across macrocyclic rings and of sets of such hydrogen-bonding pairs. The geometry of sharp ring turns is such that hydrogen-bonding pairs will have shortest path lengths of 5 ring atoms or greater, but, often, such interactions involve much larger spans. Figure 2 shows the structure of a 14 residue cyclic peptide with a disulfide bridge (the sunflower trypsin inhibitor from the PDB complex 4KEL). Five hydrogen bonds are labeled A–E (yellow dashed lines, with distances in Å), and the respective ring atom numbers along the main macrocycle are indicated (a total of 42 atoms are in the ring, excluding the disulfide bridge).

Using *only* the topology of a macrocyclic structure, it is possible to identify sets of mutually consistent hydrogen bond pairs. In the example, the triplet of A-B-C has the characteristic that on one side of the ring, the topological indices increase by 2 and 4 moving across the triplet, but on the other side, the the indices *decrease* by exactly 2 and 4. The ForceGen method identifies all such topologically compatible triplets of trans-annular hydrogen bonds, accounting for the complexities of ring numbering induced by bridges. For each such triplet, an initial structure is generated with forces applied to yield the preferred trio of hydrogen bonds. Those with the most favorable initial average hydrogen-bond distances are retained for ring conformer elaboration. In addition, ring conformer search is carried out *without* any hydrogen-bonding triplet constraint, to avoid turning a heuristic search ploy into a hard and fast assumption. The results of these independent ring conformer explorations are combined prior to torsional elaboration of exocyclic components (additional details can be found in the Methods section).

In Fig. 2, the crystallographic pose of the ligand (green) is shown with the best exemplar from a pool of 1000 conformers generated using ForceGen’s thorough search mode (cyan). The RMS deviation (beginning from a memory-free starting point) was 1.6 Å (ring RMSD of 0.8 Å). Structures of the complexity seen in Fig. 2 are not always tractable using even the most exhaustive search implemented within ForceGen. Also, it may be important to obtain a more complete and representative ensemble of what is relevant in the system under study. Here, we show a case study using Aureobasidin A (a depsipeptide antifungal with a 27-atom macrocyclic ring), where sparse distance and torsion constraints from NMR are used to enhance sampling of biologically relevant conformational space.

The results presented here exhaustively characterize ForceGen’s performance on the largest non-macrocycle and macrocycle benchmarks currently available. ForceGen is a general method whose performance, both in terms of speed and quality, represents a significant advance over existing conformer generation approaches, including performance on non-macrocycles but particularly on macrocyclic molecules. Extensions of the method are natural, whether by adding new physical movements to explore energetically viable motifs or by incorporating additional data such as NMR constraints.

ForceGen is implemented within the Tools module of the Surflex Platform, now in version 4.4.

## Methods

Where possible, data were collected to support fair and direct comparisons between the methods reported here and widely used alternatives. Every effort has been made here to ensure that the curated data fairly represents the structural data underpinning other published reports, and great care has been taken to remove *all* memory of 3D coordinates prior to generating initial 3D structural models and proceeding with conformational elaboration. Note that all non-ForceGen performance data were taken from the cited literature, where experts applied the respective methods to data sets specifically prepared for utilization by those methods.

### Molecular data sets

The results in this work were derived from the data summarized in Table 1. The 2859 molecule Platinum Set has similar characteristics to those used for development and validation of several conformer generation approaches (e.g. OMEGA and ConfGen [7, 8]), but it is much larger, and it has been very carefully curated in a manner unbiased toward any particular algorithmic approach [19]. It contains 29 macrocyclic ligands (about 1%), but the remaining 99% span a wide variety of biologically relevant molecular space.

The 30 ligand set from Chen and Foloppe [20] became an early and influential benchmark, though its size sharply limits its ability to distinguish performance of different methods with statistical support. The 182 macrocycle ForceGen Set matches closely with the Foloppe Set in terms of molecular complexity, but the sixfold increase in size makes it possible to demonstrate significant differences between alternative approaches. The 150 macrocycle set (the “Shelley Set”) that was curated for the MacroModel study [11] contains generally larger molecules than seen in the ForceGen Set (by about ten heavy atoms) and bigger macrocycles (ring sizes increased by roughly three atoms). The set is also characterized by a larger fraction of peptidic macrocycles where trans-annular hydrogen bonding is an important structural feature of low-energy conformations. The recently reported 208 macrocycle set (the “Prime-MCS Set”) is similar in many respects to the Shelley Set, though the Prime-MCS Set is less skewed toward very large molecules than the Shelley Set.

The details of the curation approach for the Chen/Foloppe and ForceGen sets are provided in the original ForceGen paper [18]. The Platinum Set was downloaded and used unmodified (Version 2017_01 from http://www.zbh.uni-hamburg.de/platinum_dataset) [19]. The Shelley Set was comprised of 67 PDB macrocyclic ligands in complex with cognate proteins along with 83 small-molecule CSD ligands. The PDB ligands were provided in Supplementary Material, sensibly protonated, in an SDF file and were converted to SYBYL mol2 without modification. The CSD ligands were obtained directly from the CCDC using reference codes provided in Supplementary Material. These were obtained as SYBYL mol2 files and had protons added automatically, as appropriate for physiological pH using the normal Surflex-Tools heuristic protonation procedure.

The Prime MCS Set was comprised of 130 CSD ligands, 60 PDB ligands (a subset of the Shelly Set), and 18 so-called “BIRD” PDB ligands (the PDB “Biologically Interesting Molecule Reference Dictionary”). The CSD portion was prepared exactly as with the Shelley Set; the 60 ligands from the Shelley Set were used as prepared within that set, and the 18 BIRD ligands were prepared from PDB files, by converting to mol2 files automatically (using the Surflex-Dock “grindpdb” procedure), with manual review of the final structures.

Taken all together, this collection of macrocyclic structural data is the most comprehensive to be the subject of a single study. In total, there are 431 distinct macrocyclic ligand structures spanning ring sizes of 9–50 atoms, total macrocyclic ring system sizes of 9–66, non-ring rotatable bonds of 0–36, rotatable macrocyclic ring bonds of 4–52, and total flexibility of 6–66 (the sum of macrocyclic and exocyclic rotatable bonds).

### Methodological details

The ring bending and twisting procedures were presented in detail in the original paper [18] and will be briefly summarized here. The new features involving flipping movements, hydrogen bonding, and NMR constraints will be covered in more detail.

#### Ring bending

The ring bending procedure have five steps:

1. *Identify ring systems* Given a single reasonable 3D conformer for a molecule, ring systems are identified where all bonds between atoms of the ring system are part of rings of size three to eight.
2. *Identify ring bends*: For any pair of atoms within a ring system, it will be used as a ring bend if the following three conditions hold:
  - *Not-connected* Each ring bend pair must not be directly bonded.
  - *Non-planarity* At least one atom of a ring bend pair must be part of a non-planar ring.
  - *Bridged or fused rings* The pair must not cross a bridged ring atom or a ring fusion.
3. *Identify LHS and RHS sides for bends* For each ring bend, we identify the “sides” of the bend and arbitrarily call the smaller of the two the right-hand-side. The atoms of the ring system form the RHS and LHS sides, and their pendant substituents are noted.
4. *Iterate over bends* For each ring bend, we will do the following:
  - *Make a bend* Centroid locations are computed for the LHS and RHS ring system atoms. The torsion angle is computed using the RHS centroid, the ring bend atom pair as the axis, and the LHS centroid as the last position. A rotation around the axis is made for the RHS atoms and their pendant groups such that the ring is bent opposite to its existing configuration (see Fig. 1). Neither the LHS atoms/substituents or the axis atoms/substituents are moved.
  - *Relax the bend* The atoms of the ring system are “pinned” using a quadratic positional penalty to prevent reversion (unbending) and the conformer is minimized.
  - *Finalize the bend* The pinned atoms are released, and the conformer is minimized again.
  - *Check quality and add to ring conformers* If the resulting conformer has not inverted any specified configurations, falls within an energy window of the current minimum, and is non-redundant based on RMSD of ring system atoms, it is added to a growing list of ring conformers.
5. *Termination and Iteration*: This process iterates through all ring bends repeatedly until either no new ring conformers are found or a maximal number of rounds are completed.

#### Ring twisting

For macrocyclic systems, in addition to bends, a twisting movement is also applied using a similar procedure to the one used for bending. The application of macrocycle twists occurs after Step 4 in the above procedure. Any single bond within a ring whose smallest enclosing ring size is nine or greater will be twisted. Each such twist consists of the two central bonded atoms (e.g. atoms 2 and 3 in Fig. 1 along with the connected ring atoms (atoms 1 and 4). Such bonds are to be twisted, as follows:

1. *Pin the non-moving atoms* Atoms 1, 2, and 3 of the torsion are pinned with quadratic positional constraints.
2. *Rotate the other atom* A series of positions for atom 4 are identified that represent rotations around the 2–3 axis. For each of these positions, a quadratic position constraint is set, and a copy of the parent conformer is minimized subject to the pinned positions.
3. *Finalize the twists* The pins are released, and the twisted conformers are minimized.
4. *Repeat with other end* The preceding steps are redone, but with atom 1 moving instead of atom 4.
5. *Check quality and add to ring conformers* For the resulting conformers that have not inverted any specified configurations, fall within an energy window of the current minimum, and are non-redundant based on RMSD of ring system atoms, they are added to a growing list of ring conformers.

Pinning the trio of atoms during each twist holds just a part of the macrocycle in place, but it allows the remaining atoms to move so as to adapt to the forced rotation of the fourth atom. In Fig. 1, only the two closest unpinned carbon atoms to Atom 4 move significantly, with the remaining atoms reacting very little to the perturbation.

#### Sub-cycle flipping

The flip physical motion identifies nearly co-linear macrocyclic ring bond pairs that are rotatable (marked by red arrows in Fig. 2). For each conformer to be considered for a flip, the following procedure is carried out:

1. *Identify relevant pairs of macrocyclic bonds* All pairs of macrocyclic bonds are identified where:
  - *Bridged or fused rings* The pair must not cross a macrocycle bridge.
  - *Distant enough*: The mid-points of each bond (arbitrarily labeled LHS and RHS) are calculated, as is the distance between the two. If the distance is at least 4.0 Å, then the pair is considered further.
  - *Directionally compatible* Three normalized vectors are calculated: (1) along the LHS bond ($\hbox{V}_1$), (2) along the RHS bond ($\hbox{V}_2$), (3) and between the mid-points previously calculated ($\hbox{V}_3$). If $min((V_1 \cdot V_2),(V_1 \cdot V_3),(V_2 \cdot V_3)) > 0.3$, then the pair is considered further. Note that this is a weak definition of co-linearity, allowing for angular deviations from co-linearity of about 70 degrees.
2. *Make the flip* For each bond-flip pair:
  - *Identify atoms to be flipped* All atoms between the ends of the two bonds are identified.
  - *Flip the atoms* These atoms are rotated 180 degrees around the axis between the midpoints of the two bonds.
  - *Pin the ends* The end atoms of the flip are pinned using a quadratic position constraint.
  - *Minimize* The sub-cycle flipped conformer is minimized, and the positional restraints are released. If the resulting conformer has not inverted any chiral centers and is within the current energy window (or forms a new lower bound), it will be retained.

A very similar procedure is used to flip bridging components that connect different sides of a macrocycle. In this situation, it is very likely that chiral centers (especially those that occur at the bridging atoms) will be inverted. Rather than enforcing a positional constraint as with the flipping procedure, all chiral configurations are enforced with standard improper torsion terms, minimization is done, and the chiral constraints are removed. If the resulting conformer has not inverted any chiral centers and is within the current energy window, it will be retained.

#### Hydrogen bond exploration

Cyclic peptides and related macrocycles often exhibit multiple trans-annular hydrogen bonds that stabilize a family of low-energy conformations. While it is possible to happen upon such configurations through the movements just described, in general, the search times to reliably find these configurations will be large. As seen in Fig. 2, the topological features that are seen in peptidic ligands are both easy to identify and can provide strong constraints on the combinations of hydrogen bonds that may be profitable to explore.

1. *Identify relevant hydrogen-bonding pairs* All pairs of macrocyclic hydrogen bond pairs are identified:
  - *Macrocycle parsing* The macrocycles within a molecule are identified.
    i. *Macrocyclic bonds* A pair of atoms whose shortest connecting path contains nine atoms or more is labeled as a macrocyclic bond, and the atoms are marked as being part of a macrocycle.
    ii. *Macrocyclic systems* From each macrocyclic atom that has not been labeled with a system number, all atoms that are connected by bonds that are part of rings are iteratively identified. All such atoms are labeled with the same macrocyclic system number. The procedure identifies all macrocyclic systems and separately labels each one.
    iii. *Macrocyclic bridges* Bridges within macrocyclic systems are identified topologically. The simple case is where two atoms in a macrocyclic system each have three bonds connecting them to three atoms that are all labeled as being part of the same system (as seen in the 4KEL example of Fig. 2). More complex cases involve a small aromatic ring or ring system that mediates a bridge, and these are also identified. By convention, the shortest path connecting bridging atoms is called the bridge.
  - *Hydrogen bond pair constraints* Donor/acceptor pairs that are part of the same macrocyclic system are identified, subject to these constraints:
    i. *Do not cross a bridge atom* The shortest non-bridging path between the atom pair must not include a bridge atom. For example, in Fig. 2, the proton attached to ring-atom 3 is not paired with the carbonyl oxygen attached to ring-atom 19. This is not to say that such interactions cannot exist; rather, they simply are not part of the set of explicitly explored interactions.
    ii. *Distant enough to make a turn* If the shortest non-bridging path between a donor and acceptor (including the ends) is at least eight, they are added to the list of h-bond pairs to explore.
2. *Identify hydrogen-bond triplet sets* Given the set of hydrogen bonding pairs just identified, those groups of three that are capable of forming topologically compatible simultaneous h-bonds are enumerated, with the constraints described in the Introduction.
3. *Generate constrained macrocycle alternates* The preceding steps may identify a very large number of possible triplets to explore. For example, the 4KEL ligand (see Fig. 2) has 39 triplets that are identified after meeting all of the preceding topological constraints.
  - For each triplet, a constrained minimization is carried out where the triplet of h-bonds in question is constrained with a quadratic penalty to have an inter-atomic distance of 2.0 Å or less.
  - The set of minimized triplet alternatives is sorted based on mean h-bond triplet distance (low to high), with the top 8 retained for explicit exploration.
  - For each such remaining alternative, a conformation is generated beginning from zeroed coordinates, but while making use of the triplet of h-bond distance constraints.
  - This set of alternative h-bond triplet constrained conformational starting points is added to the original *h-bond agnostic* conformation. All are then subjected to the normal ForceGen ring search procedure, with the agnostic starting point having no fixed h-bond constraints and with the h-bond triplet alternatives being searched with the constraints in place.
  - Based on energy, the best ring alternatives from this procedure are retained, and the exocyclic torsional elaboration proceeds using the normal procedure.

This strategy for exploring macrocyclic hydrogen bond networks can increase the computational cost by nearly a factor of 10. However, all of the alternative macrocyclic explorations are done in parallel, with each individual sub-exploration also benefiting from multi-core parallelism. Such cases remain the most computationally expensive to explore, as they combine the largest molecular sizes, largest ring sizes, and also incur the multiplicative cost of searching alternate constrained starting points.

#### NMR constraints

Utilization of experimental data derived by NMR is natural within the ForceGen algorithm, and it uses the same computational machinery as used for the hydrogen bond networks just described. Specification of distant constraints is done as follows: 
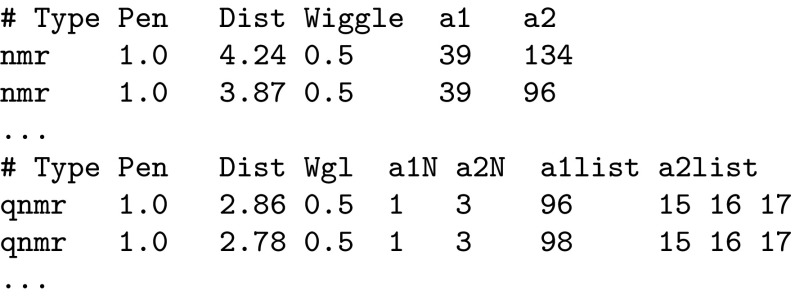


Distance constraints can be specified either between specific protons (the “nmr” type) or between symmetry-related groups of protons (the “qnmr” type), where the centroid of the specified atoms serves as the point from which distances are calculated. The latter is a common approach for handling degenerate proton resonances, implemented in widely used NMR-based conformational analysis software such as CYANA [22]. In each case, a penalty value (kcal/mol/$\hbox{A}^2$) defines the strength of the positional constraint, whose ideal value is specified by the “dist” parameter. Free movement is allowed up to the value specified by “wiggle” beyond which a quadratic penalty applies. This creates a simple square-welled, continuous, and differentiable penalty function that accommodates experimental noise.

Specification of torsional constraints is very similar: 
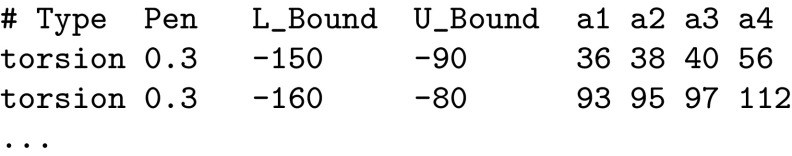


Again, a smooth square-welled penalty is defined, with a value of zero between the specified bounds and increasing quadratically beyond those bounds. Conventional definitions, as commonly used within the peptide NMR community, of $\phi$ and $\psi$ angles are employed.

The option “-molconstraints *constraint_file*” passes the constraints into the ForceGen procedure from the command line. The constraints are used both for initial 3D structure generation as well as during the entirety of conformational search.

Conformer ensembles generated under one set of NMR constraints (or none at all) may be profiled against that set of constraints or another set using the “profile” ForceGen procedure. The profile yields information about the energetics of each conformer, expressed as both the MMFF94sf energy as well as the energy of constraint violation (the “violation energy”). It also counts discrete violations per conformer subject to specific thresholds. In addition to profiling conformers, the procedure produces a profile of each NMR constraint that summarizes the extent to which it is violated in terms of frequency and magnitude in a fashion similar to CYANA [22]. This can be helpful in identifying incorrectly assigned NMR peaks by making use of subsets of possible constraints.

The profiling procedure optionally takes a conformer as an argument against which to measure RMSD (automorph and alignment corrected) in cases where there may be some orthogonal experimental information about the preferred conformation of the molecule under study. 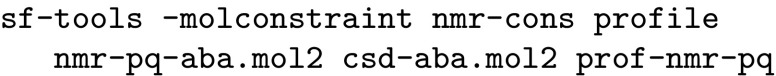


The preceding command generates a profile for the given conformer ensemble (“nmr-pq-aba.mol2”) against the NMR constraints in “nmr-cons” while measuring closeness to the CSD crystal structure “csd-aba.mol2” with the resulting conformer and constraint profiles (tab-delimited text files) being prefixed by the last argument.

#### ForceGen search modes

The machinery for torsional sampling has been further optimized for computational speed and parallel computations, and parameter sets have been developed for different accuracy/speed trade-offs. ForceGen offers seven user-selectable modes of conformational search:

*-pfastf* for preparing very large compound databases (50 conformers max)
*-pscreen* the preferred mode for preparation of all but the very largest databases for virtual screening (50 or 120 max conformers depending on ligand flexibility)
*-pfast* an alternative to -pscreen where a reduction of the total number of conformers per molecule is important (50 conf. max)
*-pgeomf (default)* appropriate for fast geometric sampling of ligands (250 conf. max)
*-pgeom* standard search mode, for geometric studies, including macrocycles (250 conf. max)
*-pquantf* for preparation of molecules in affinity prediction workflows (1000 conf. max)
*-pquant* thorough search mode, for more accurate preparation of molecules (including macrocycles) in affinity prediction workflows (1000 conf. max)

### Experimental NMR data for Aureobasidin A

Aureobasidin A (AbA) served as a case study for employing ForceGen’s NMR constraint functionality. All AbA NMR data were acquired at 25 degrees Celsius in $\hbox{d}_6$-DMSO. In full agreement with the original report [23], AbA exists in solution as two slowly exchanging forms: trans-Proline and cis-Proline conformers. Because the exchange is very slow on the NMR timescale, both forms can be analyzed as independent molecules [23]. In this work, we focused on trans-Proline conformation of AbA because it has been more extensively studied, and there is a single-crystal X-ray structure available for this form [24].

#### Resonance assignment and constraint generation

All isotropic NMR data were acquired on Varian 600 MHz VNMRS instrument equipped with a 3mm triple-resonance cryoprobe. NMR resonance assignment was conducted using conventional NMR approach utilizing ^1^H, ^1^H-^1^H COSY, ^1^H-^13^C HSQC, ^1^H-^13^C HMBC and ^1^H-^1^H ROESY data. NMR sample contained 7mg of AbA in 0.2ml of $\hbox{d}_6$-DMSO in a 3mm NMR tube. The parameters were as follows: ^1^H-^1^H COSY (4 scans, 400 indirect increments), ^1^H-^13^C HSQC (multiplicity-edited, 8 scans, 180 indirect increments), ^1^H-^13^C HMBC (8Hz optimized, 32 scans, 240 indirect increments), ^1^H-^1^H ROESY (ROESYAD, 80 ms mixing time, 16 scans, 400 indirect increments).

NOE distance restraints were generated from the ROESY data mentioned above. The goal of this work was to utilize restraints that were relatively easy to extract from experimental data. Typically, most peptide NOE cross-peaks have multiple possible assignments due to significant ^1^H resonance overlap, especially for the side chains. In the case of AbA, the situation was exacerbated by the presence of two forms (trans-Proline and cis-Proline), which significantly increased the degree of resonance overlap and, correspondingly, the degree of NOE assignment ambiguity. The conventional approach to resolve the ambiguity would consist of iterative refinement of the conformational structural ensemble and the NOE assignments resolvable using those structures [22]. However, such an approach is both time-consuming and potentially biases the conformational ensemble. In this work, we intentionally used only restraints generated from NOE cross-peaks with unambiguous assignments, which amounted to a total of 20 out of more than 200 individual NOE cross-peaks. Unambiguously assigned NOEs cross-peaks were integrated using MestreNova software, and their integrals were converted to interatomic distances by a conventional approach using NOEs between protons with a fixed distance for calibration [22].

$\Phi$ torsion angle restraints were generated from the $^3J_{\mathrm{HNHA}}$ coupling constants measured in $^1\hbox{H}$ spectra. There are only 3 NH protons in AbA, and all of them showed relatively large couplings (Phe3 9.9Hz, Ile6 8.0Hz, Leu8 7.9Hz), allowing the use of a generalized Karplus equation [25] to generate torsional restraints. Additional allowances were added to the restraints to account for imperfect experimental data and uncertainties in the Karplus equation parameterization.

#### Residual dipolar coupling (RDC) measurements and analyses

Anisotropic NMR data (RDC measurements) were acquired on Bruker 500 MHz Avance IIIHD instrument equipped with a 5mm CPP TCI cryoprobe. RDC measurements were performed using a stretched gel methodology (detailed in [26, 27]). ^1^H-^15^N and ^1^H-^13^C RDCs were measured as a difference between respective one-bond ^1^H-^15^N and ^1^H-^13^C coupling constants in the isotropic and anisotropic (stretched gel) environments. ^1^H-^15^N one-bond coupling constants were measured in the $^{15}\hbox{N}$ dimension of ^1^H-^15^N HSQC spectra (coupled, 90Hz optimized, 76 scans, 122 indirect increments). ^1^H-^13^C one-bond coupling constants were measured in the $^{13}\hbox{C}$ dimension of ^1^H-^13^C HSQC data (coupled, 145Hz optimized, 16 scans, 1108 indirect increments). Only backbone (NH and $\hbox{C}\alpha \hbox{H}$) RDCs were used for validation; this simplified the analysis by eliminating the need to account for RDC averaging in flexible side chains.

RDC data were used for an orthogonal validation of generated conformational ensembles and were not used as restraints. For every structure in the ensemble, a single value decomposition (SVD) analysis was utilized to calculate the alignment tensor and the Q-factor, which measures a quality value if a correlation exists between the experimental and back-calculated RDC values [28].

### Computational procedures and statistical analysis

The results reported here were generated using Surflex-Tools version 4.411. The results were generated through zeroed-coordinate conformer randomization in standard search mode, as follows (shown for the ForceGen Macrocycle Set): 
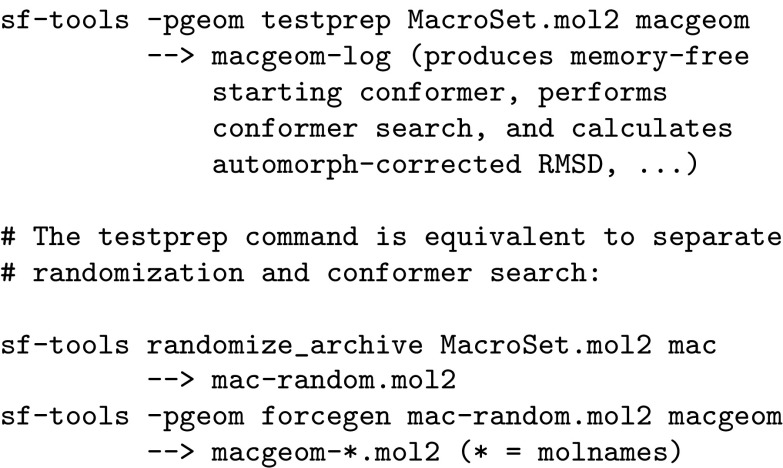


RMS deviations were done for each resulting conformer pool by identifying all molecular symmetries, then applying the rigid body alignment transform to each conformer so as to minimize the RMSD against the crystallographic one under all identified symmetric self matches. The minimum such RMSD value (for non-hydrogen atoms) is the value reported for each ligand. RMSD of heavy atoms corrected for molecular automorphism is standard in evaluations of docking calculations and for conformer generation.

In addition, for ligands containing macrocycles, calculations were made for the RMSD of the atoms within the macrocycle rings. For all ring bonds, the ring system comprised of enumerating all connected ring bonds is identified. Given such a ring system, the shortest path between any two bonded atoms is calculated. Where a ring bond exists whose shortest path is nine or greater, the ring system is said to be macrocyclic. The *size* of that macrocycle is taken to be the smallest of the various macrocyclic paths within the ring system. For molecules with multiple macrocyclic ring systems, the largest such size is reported as the macrocycle ring size of the overall molecule.

**Table 2:** Summary of key performance characteristics for ForceGen and other methods on the Platinum Diverse Dataset, with bold values for best same-mode performance within 0.05 Å (mean RMSD), within 0.2 Å (95th percentile RMSD), a factor of two (median and mean times), and 4 percentage points (success rate at 1.0 Å RMSD)

Method	Mean	95th Pctl.	Median	Mean	Success at	Mean	Max
	RMSD (Å)	RMSD (Å)	Time (s)	Time (s)	1.00 Å (%)	Pool Size	Pool Size
Screening mode							
FGen -pfastf	0.70	**1.56**	**0.30**	**0.62**	78	39	50
FGen -pscreen	**0.63**	**1.42**	**0.48**	**1.07**	**84**	62	50/120
OMEGA	**0.67**	1.7	2	**2**	**80**	34	50
iCon	0.72	1.8	5	5	76	35	50
RDKit-DG	0.77	2.3	4	5	71	50	50
cxcalc	0.87	2.1	5	6	63	48	250
ConfGenX	0.69	1.7	9	13	77	39	50
MOE	0.75	2.2	62	158	76	30	50
Accurate mode							
FGen -pgeomf	**0.58**	**1.27**	**0.61**	**1.53**	**87**	156	250
FGen -pgeom	**0.55**	**1.22**	**0.88**	**2.89**	**89**	170	250
OMEGA	**0.57**	**1.4**	**2**	**3**	**87**	118	250
iCon	**0.60**	1.5	5	5	84	123	250
RDKit-DG	0.63	1.5	17	22	82	250	250
cxcalc	0.73	1.8	17	21	72	227	250
ConfGenX	**0.58**	**1.4**	13	14	**86**	160	250
MOE	0.64	1.6	61	153	83	77	250

Values for alternative methods were taken from [19] all using the default operation modes of the best variants: OMEGA, iCon, RDKit-DG, ConfGenX, cxcalc, and MOE-Stochastic (sorted in rough order of speed)

In comparisons of performance between methods, where data have been available linking specific molecules to performance values, paired t-tests have been used to calculate p-values for superiority of one method over another. Where only accumulation curves or unlabeled collections of performance values have been available, and where two distributions clearly favor one method over another, Kolmogorov-Smirnov (KS) tests have been done to calculate p-values (these are based on the maximal difference in cumulative distributions of performance values for two methods). KS tests are less sensitive than paired t-tests, and for small data sets, the difference in cumulative probability must be quite large.

For example, for the Chen/Foloppe Set (30 examples), a KS test requires a maximal difference of roughly 0.35 at p = 0.05 (i.e. a 35 percentage point difference in RMSD success rates). For moderately sized data sets (200 examples), a 14 percentage point difference is sufficient. For large sets such as the Platinum Diverse Set (2859 examples), a performance difference of just 4.3 points is sufficient to distinguish significant differences at p = 0.01.

Additional details about the data sets, computational procedures, and about software availability are available at www.jainlab.org.

## Results and discussion

ForceGen was introduced with analysis of both 3D structure generation and conformer generation using five data sets, but comparison to other approaches was limited by the size of available benchmarking data sets and the breadth of available comparative data to recent versions of widely used methods [18]. Here, we will present updated performance, both for accuracy and speed, including much more extensive direct comparative analysis. The recent paper introducing the Platinum Diverse Set included detailed benchmarking for several widely used methods [19], and it will be used for comparative analysis on non-macrocyclic conformer search performance.

For macrocyclic performance, the ForceGen Set will be used to characterize performance gains attributable to the new search strategies, and the other three macrocycle sets will be used for comparison to other methods. Last, Aureobasidin A (AbA) will be used as a case study in the utilization of NMR distance and torsional constraint data for the generation of macrocyclic conformer ensembles.

**Fig. 3:**
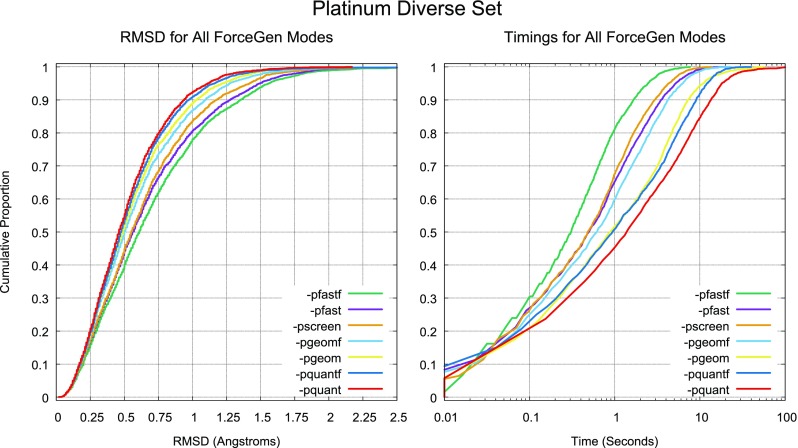
Accuracy and timing for all seven ForceGen 4.4 Modes for the Platinum Diverse Set of 2859 PDB ligands

### Platinum diverse set

The Platinum Diverse Set was specifically curated for evaluation of conformer generation by systematic and automated processing of protein-bound ligand structures from the PDB, resulting in 2859 ligands in the 2017 revision [19], which limits the over-representation of molecular scaffolds. Proportionately, it contains few macrocyclic compounds (just 1%), and those present are less complex than those in the macrocycle-focused sets (see Table 1).

Table 2 summarizes results for ForceGen and the most successful of the variants within each of six programs reported in Friedrich et al. [19]. Multiple parameterizations of several programs were run, generally with the default methods performing best. The data excerpted here represents a best-case for nearly all programs and always at least a very good case of program performance, but many details in the original study may be of further interest to readers [19]. Performance for default parameterization of the following program variants are shown in Table 2 (force fields used for conformer minimization are in parentheses): OMEGA (none), iCon (MMFF94s), RDKit-DG (UFF), cxcalc (Dreiding), ConfGenX (none), and MOE Stochastic (MMFF94x). The following variants performed less well and will be be discussed further in the context of non-macrocyclic molecules: MOE LowModeMD, MOE Import, and ConfGen.

The Table is split into results for screening-mode conformer preparation (top half) and accurate-mode (bottom half, e.g. for pose prediction). Bold values represent the top performing methods within each mode. The *-pscreen* variant makes use of a ligand-dependent variable-sized final pose pool (50 or 120 depending on flexibility), which resulted in slightly larger final conformer ensembles (roughly 60 compared with roughly 40 for the other methods). However, for this small increase in mean pool size, performance across all criteria was the highest. Performance for the ForceGen *-pscreen* approach was comparable to the other methods from the *accurate-mode* tests.

For the screening mode test, ForceGen produced the fastest performance (both the *-pfastf* and *-pscreen* variants). OMEGA was the fastest of the other methods, though still 2–4 times slower than the ForceGen screening modes. For large collections of small molecules, simple parallelism across independent computing nodes is trivial to implement and is essentially perfectly scalable, so speed differences of a few fold should not be considered to be terribly important. However, speed differences of 5–10 or more have serious practical consequences in terms of either time or cost of computing resources. So, while, for example, ConfGenX performed reasonably well, its relative speed may be limiting.

**Table 3:** Summary of ForceGen calculation speed using different hardware and core/thread combinations, with throughput calculated assuming molecule-level parallelism in the 36-core 1-thread scenario (36 simultaneous single-threaded jobs)

Method	N cores	N threads	Median time (s)	Mean time (s)	N jobs	Molecules per hour	Molecules per day
FGen -pfastf	36	36	0.30	0.62	1	5806	–
FGen -pfastf	4	8	0.52	0.93	1	3865	–
FGen -pfastf	36	1	0.59	1.35	36	96,000	2,304,000
FGen -pscreen	36	36	0.48	1.07	1	3364	–
FGen -pscreen	4	8	0.92	1.69	1	2133	–
FGen -pscreen	36	1	1.28	3.03	36	42,772	1,026,534
FGen -pgeomf	36	36	0.61	1.53	1	2352	–
FGen -pgeomf	4	8	1.24	2.61	1	1382	–
FGen -pgeomf	36	1	1.62	4.45	36	29,123	698,966
FGen -pgeom	36	36	0.88	2.89	1	1246	–
FGen -pgeom	4	8	2.00	6.32	1	570	–
FGen -pgeom	36	1	3.01	15.11	36	8577	205,850

With respect to accuracy, based on mean RMSD and success rate at 1.0 Å RMSD, the two ForceGen screening modes, OMEGA, and ConfGenX all performed well, with iCon, RDKit, cxcalc, and MOE forming a second tier. Many researchers focus on median or mean RMSD values to characterize conformer generation performance, but this has two limitations. First, for good methods, mean RMSD begins to push the limits of experimental uncertainty in ligand coordinates in X-ray structures of bound ligands. Second, it is arguably less important to know how well a method performs on the best half of one’s data than on, say 95% of the data one is likely to encounter. Table 2 provides the RMSD values at the 95th percentile ($\hbox{RMSD}^{95}$) for each method. The two ForceGen screening modes ranged from 1.4 to 1.6 Å, and the other methods ranged from 1.7 to 2.3 Å. Even in the fastest ForceGen search mode, 95% of molecules are expected to have a conformer within 1.6 Å RMSD of the bioactive one. The ForceGen algorithm does not seek to rapidly enumerate non-redundant conformers based on torsion libraries, which is a strategy employed by, for example, OMEGA. Rather, the ForceGen approach seeks to identify diverse, low-energy, conformers without reference to the structures of prior known ligands.

Performance in the accurate-mode tests showed a similar pattern to the screening-mode results, with the ForceGen being top-performing with respect to both speed and accuracy, especially in terms of $\hbox{RMSD}^{95}.$ Again, OMEGA was the fastest of the other methods, and, except for $\hbox{RMSD}^{95}$ performance, it was comparable to ForceGen. Of the remaining methods, ConfGenX was the best performing, though the cost in time was significant. None of the other methods produced a competitive balance of time and quality. Note that with 2859 compounds in the Platinum dataset, performance differences of between 3–4% become both practically and statistically significant at any success threshold, so considering performance at 1.0 Å, ForceGen’s 89% success in accurate-mode was clearly better than iCon, RDKit, cxcalc, and MOE.

Figure 3 depicts ForceGen accuracy and computational cost for seven user-selectable accuracy/time modes. For screening, the *-pscreen* approach (orange lines) offers a good trade-off in terms of accuracy, speed, and conformational ensemble size. For more accurate exploration of non-macrocycles for pose prediction, the *-pgeomf* option (light blue) offers faster performance than the *-pgeom* option (yellow), with only a minor reduction in accuracy. For more exhaustive search, typically applied in affinity prediction exercises, the *-pquantf* option offers a good balance between speed and accuracy. For macrocycles, the deeper ring search offered by the *-pgeom* and *-pquant* modes is important, to be discussed next.

Because ForceGen is template-free, relying only on molecular energetics to produce conformer ensembles, it may perform better on novel compounds that are not currently represented within the PDB. Template-based methods (e.g. OMEGA) base their internal ring geometries and torsion libraries in large part on the very data that comprise the Platinum Diverse Set itself, potentially overfitting the corpus of what is known. In terms of overall computational strategy, the other distinguishing feature of ForceGen is that it intentionally optimizes the diversity of conformational ensembles within a low energy window, avoiding over-representation of minor conformer variants. This manifests most directly in the robust performance of ForceGen in all search modes at RMSD cutoffs of 1.75 Å or higher (see Fig. 3).

In addition, input to ForceGen is done through standard file formats (e.g. mol2, sdf, or SMILES), without requiring any template definitions or pre-defined atom types. Molecules may be hybrids of standard amino acids, non-standard amino acids, contain organic chemical linkers, and may be cross-linked arbitrarily. The only requirement is that the molecular composition has defined parameters with MMFF94sf (currently including the most common organic structures with the following atoms: H, C, N, O, P, S, F, Cl, Br, and I).

### Timing considerations and multi-core parallelism

For non-macrocyclic ligands, databases exist that contain many millions of compounds, and combinatorial exploration of analogs is common. Consequently, scenarios for computational modeling span a large range in terms of needs for throughput. Interactive modeling, on a workstation or a laptop, might consider just a few or perhaps dozens of molecules. Large-scale preparation of databases for virtual screening may consider millions of compounds, but such calculations will typically take place on a high-performance computing cluster or in a cloud computing environment.

The timings shown in Table 3 were carried out on two very different hardware configurations. The first was equipped with dual Intel Xeon Platinum 8124M CPUs, operating at 3.00 GHz, with a total of 36 physical computing cores, each capable of running 2 threads. A comparably equipped modeling workstation with similar memory would cost roughly $10,000–$15,000 depending on graphics and storage options. This corresponds to an Amazon Web Services c5.18x-large instance, which can also be scaled for large-scale applications at a spot price of $1.159 per hour per instance. The second configuration was a highly portable Dell XPS 13 (circa 2018), equipped with an Intel Core i7-8550U CPU operating from 1.80 to 1.99 GHz, with 4 computing cores, each capable of running 2 threads.

Interactive time-scale modeling can take place on office-deployed workstations or on mobile laptop computers. This type of modeling will consider different types of questions, where the relevant time scale is seconds or minutes in the ideal case, up to perhaps an hour or two to accommodate lengthier calculations during which time another activity may be undertaken. For small non-macrocyclic molecules, typical workflows may include docking multiple variants of a scaffold followed by visualization.

For macrocyclic molecules, in addition to these cases, iterative refinement of NMR constraint data may be of interest, for example. In such cases, the number of available computing cores may far exceed the number of molecules under study, and, in all cases, the total wall-clock time from the beginning to the end of the calculation is critical for the productive use of human time. Here, maximizing CPU utilization through multi-core parallelism may have a significant practical impact on productivity. The speed with which a calculation on a *single* molecule can be completed might be quite important.

Large-scale calculations form a very different case, where conformational search of large corpora of molecules can be easily parallelized across multiple computing nodes, with essentially perfect linear speedup. Where millions of molecules are to be processed, both the speed of a single-threaded calculation and its memory footprint are important determinants of the overall cost of the calculation. Another consideration here is the ability to produce conformer pools of limited size within a specified energetic window, but which are still likely to sample conformational space well; this can become a particularly significant concern with some macrocycle search algorithms, which may require thousands or many thousands of conformers to cover space well and may or may not fall within a reasonable window of energies.

**Fig. 4:**
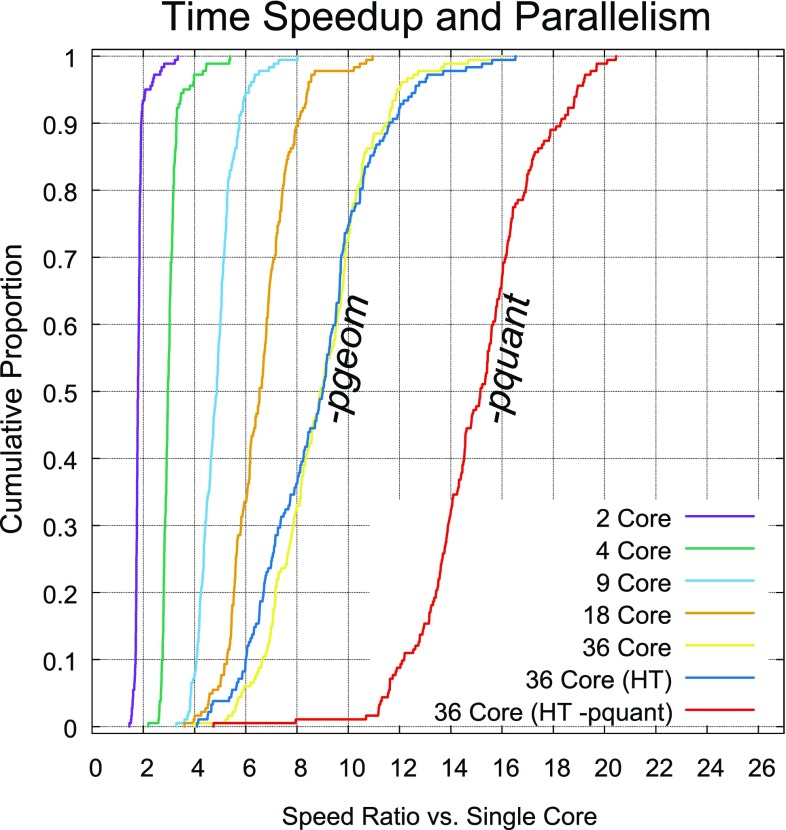
Dependence of computing time on number of parallel threads, using a 36-core AWS c5.18xlarge instance and the ForceGen Macrocycle Set to measure speed increases of multiple cores relative to a single core

Table 3 shows timing results for four parameter settings (two for screening-mode conformer search and two for accurate-mode) under three calculation protocols: (1) 36-core workstation-class hardware running a 36-thread process; (2) 4-core laptop hardware running an 8-thread process; and (3) 36-core workstation/cloud-class hardware running a single thread. In the first two situations, the computers were considered to be fully loaded, and in the last one, 36 such calculations would be run simultaneously to achieve full load without taxing either disk input/output, memory, or internal communications bandwidth.

For the interactive modeling scenarios (the first two), all conformer preparation modes yielded sufficiently high throughput to process a thousand or more molecules (screening modes) or hundreds of molecules (accurate modes) in an hour’s time. The most strenuous calculation (*-pgeom*) on a workstation, required under 3 s per molecule on average, which would support quite facile interaction in an interactive docking session. Note that the speed gain for deeper search was over fivefold (1-thread to 36-thread on a 36-core computer), compared with closer to twofold for more shallow search. Even in the mobile laptop scenario, one could make use of the *-pgeomf* mode to keep accurate conformer sampling under 4 s per molecule.

For the large-scale, possibly cloud-based, calculation scenario, maximal throughput per 36-core node was 2.3 million molecules per day, which is roughly the size of the entire ChEMBL small molecule database [29]. ForceGen *-pfastf* mode using a single computing thread is faster than the result reported for OMEGA on the Platinum Diverse Set [19], and ForceGen has comparable speed in *-pscreen* mode. Very large databases of purchasable synthesizable chemicals now exist for virtual screening. A collection of 200 AWS c5.18x-large nodes could process (*-pfastf* mode) a 500 million compound library of the complexity represented in the Platinum Diverse Set in about 1 day. The total cost would be approximately $5,000 for calculation time without accounting for network bandwidth or disk space charges (assuming spot-instance pricing circa 2018–2019).

The opportunities for parallelism within the ForceGen implementation are more numerous and more fruitful in deeper searches of more complex molecules. From Table 3, the typical speedups in a many-core/many-thread calculation ranged from just over a factor of 2 for the shallowest search (*-pscreen*) to just over a factor of 5 for the deeper search (*-pgeom*).

One important aspect of the ForceGen approach is that macrocycle conformational exploration is largely non-serial, and so multi-core parallelism can be utilized, for example, to simultaneously explore the different bends, twists, flips, and hydrogen bond formation movements of a given molecule. Figure 4 shows cumulative histograms of time speedups as the number of computing cores increase, using the ForceGen macrocycle set as a benchmark. For macrocycles, the standard depth of search is accessed with the *-pgeom* mode (left-most six curves), and the more thorough level with the *-pquant* mode (right-most curve in red). The 2-core calculation came quite close to a perfect twofold speedup across the full set of 182 molecules, but with increasing thread-count, the speedup was sub-linear.

**Fig. 5:**
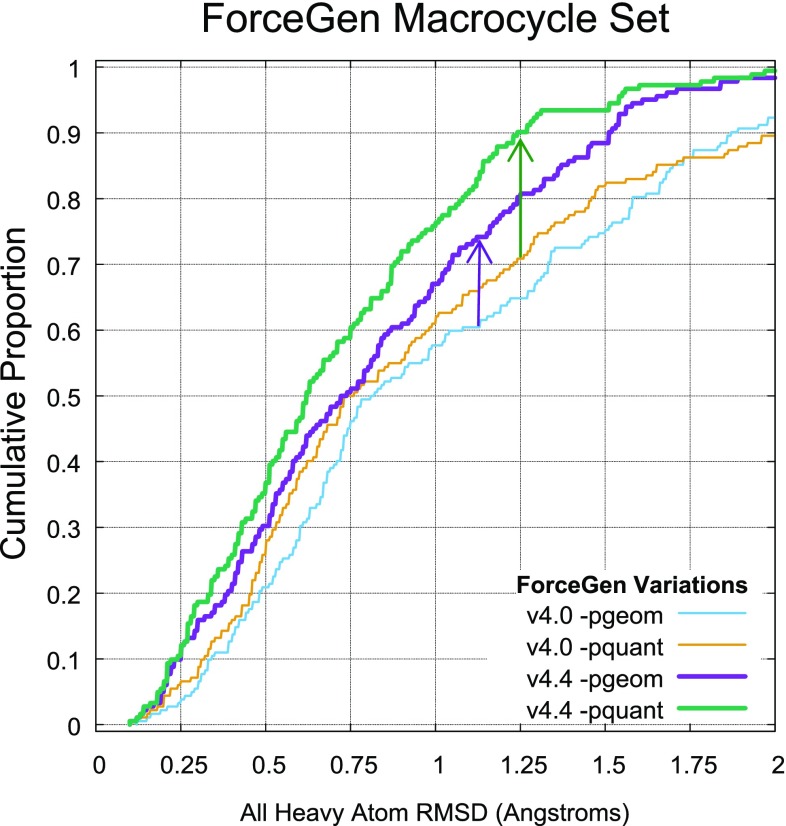
Comparison of ForceGen v4.4 (purple, and green lines) with v4.0 (thin blue and yellow lines) on the 182 macrocycle ForceGen Set as reported in the original paper [18]

Using 36 cores with a single thread per core (yellow), speed increases were typically 8–10 fold. With 2 threads per core (blue), the fraction of high core utilization increased, and overall, the mean calculation time was slightly faster with 72 threads (blue line) than with 36 (yellow line). With deeper search (the *-pquant* mode, red curve), larger gains were evident, with 70% of examples obtaining speed increases of 14–21 fold. In the results that follow, ForceGen per-molecule times, unless otherwise noted, were the result of calculations using 72 threads on a 36-core AWS c5.18x-large instance.

**Fig. 6:**
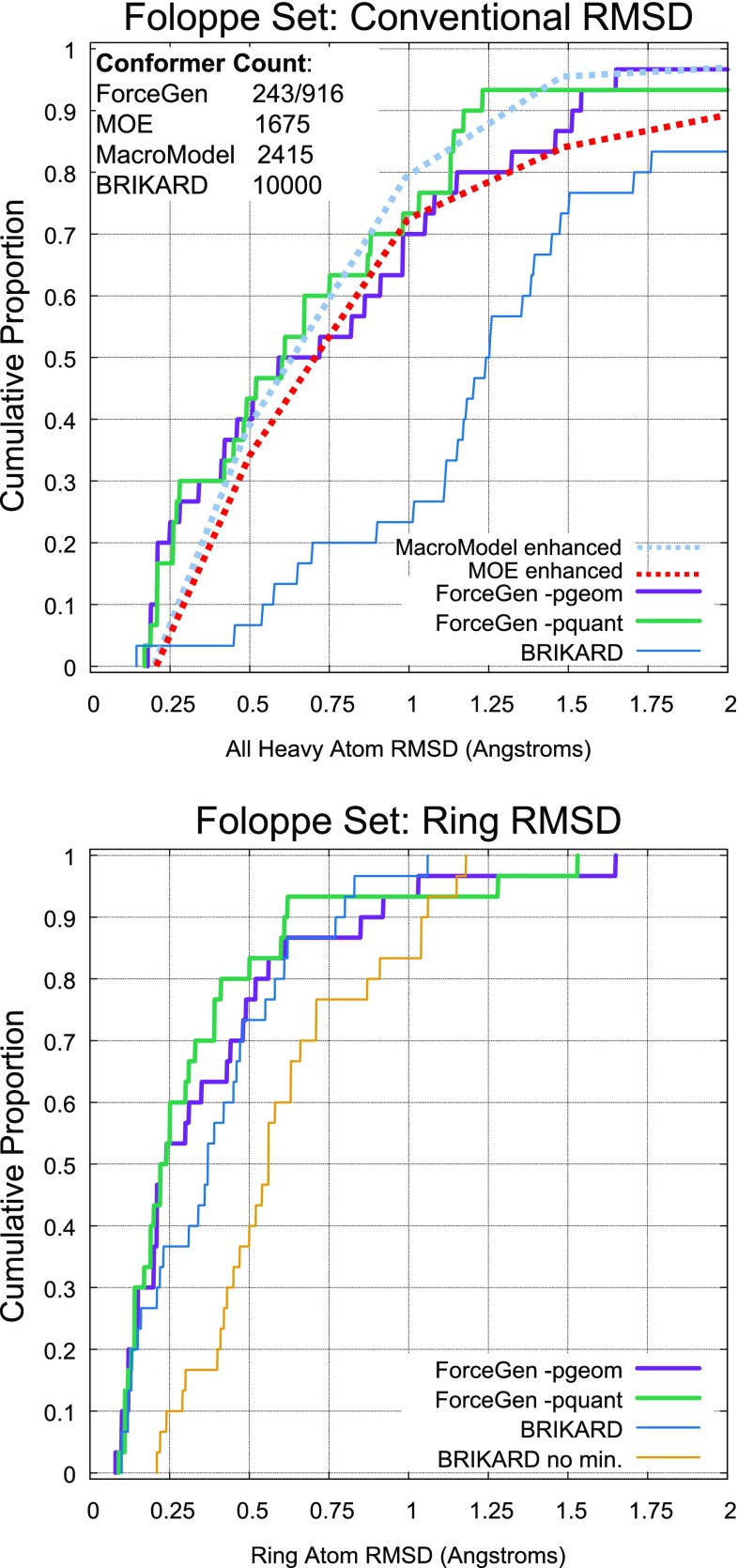
Comparison of ForceGen v4.4 (purple, and green lines) MOE, MacroModel, and BRIKARD on the 30 macrocycle Chen and Foloppe set [20]

**Fig. 7:**
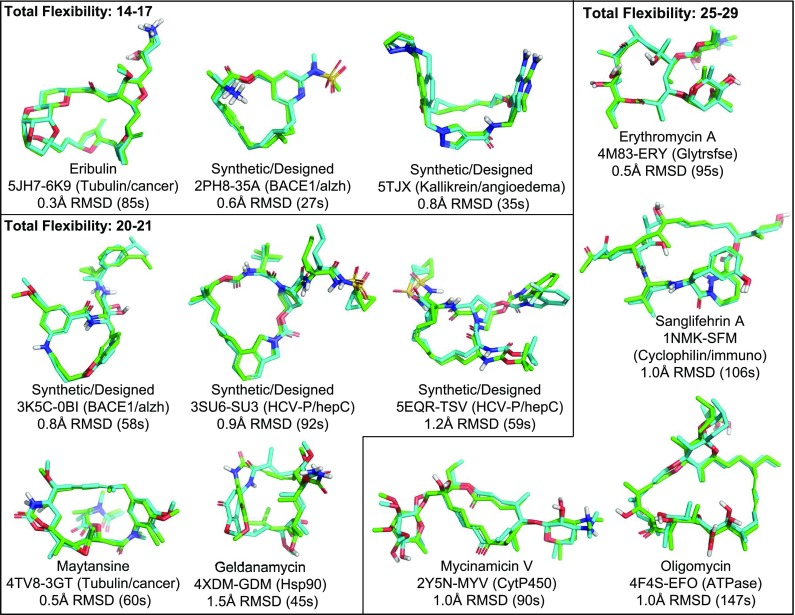
Typical examples of ForceGen v4.4 improvements in performance over v4.0 in standard (-pgeom) mode, with the average improvement in RMSD being 0.8 Å on this subset and 0.2 Å overall

### ForceGen and Chen/Foloppe macrocycle sets

The most significant changes within ForceGen version 4.4 are improvements within the macrocycle search methods, as discussed in the Introduction. Figure 5 shows the direct comparison for versions 4.4 and 4.0 on the 182 molecule ForceGen Macrocycle Set. With the new version, the standard search mode (*-pgeom*) exceeded the performance of the previous version’s thorough search mode (*-pquant*). The thorough search mode of v4.4 made a dramatic improvement over the prior version (nearly 20 percentage points at the 1.25 Å threshold). The performance gains were highly statistically significant (paired *t*-test p-values less than $10^{-10}$).

**Fig. 8:**
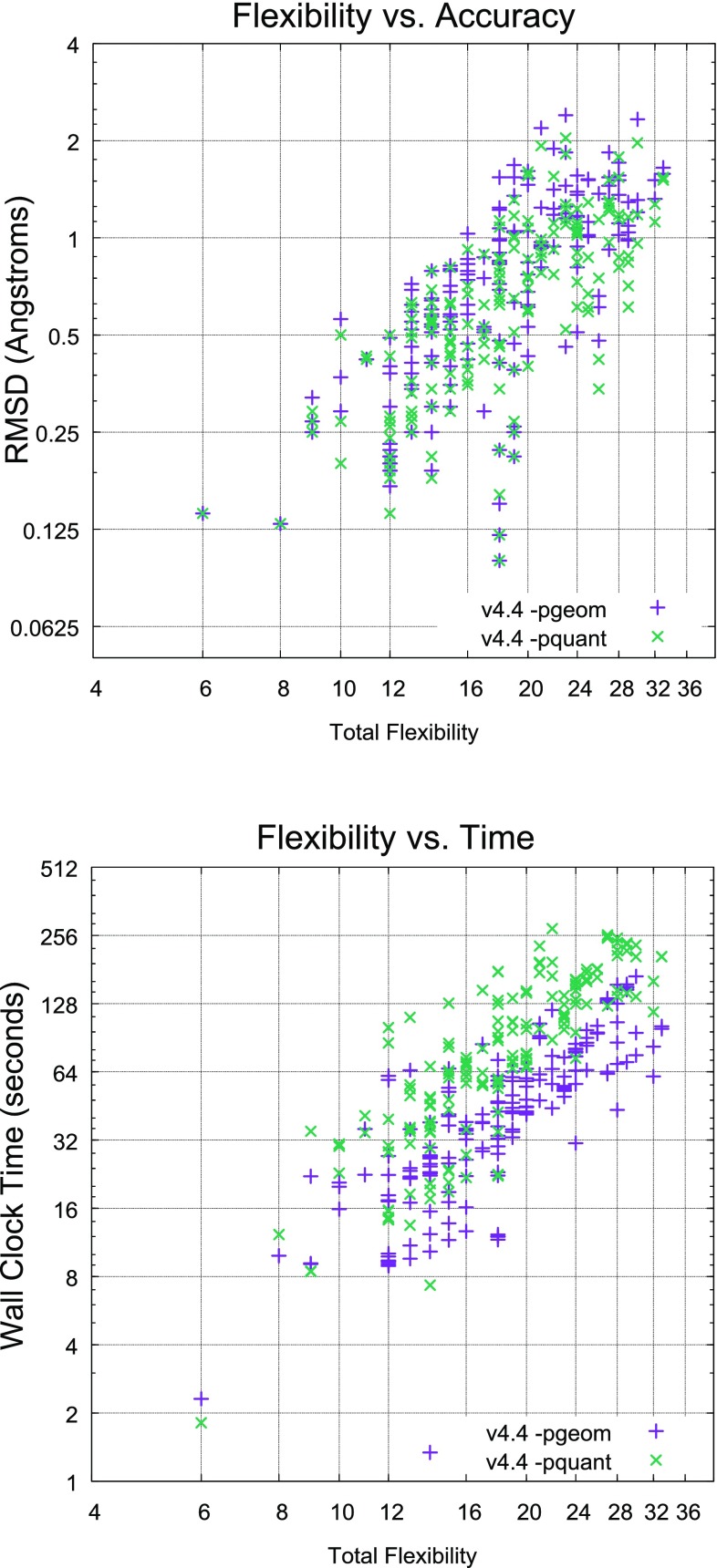
The relationship between total macrocyclic flexibility and accuracy (top) and calculation time (bottom) was close to linear

Figure 6 shows comparative performance between ForceGen and the MacroModel and MOE low-mode MD approaches on the Chen/Foloppe Set, using the enhanced parameter sets previously reported [20]. It also includes a comparison with the BRIKARD inverse-kinematics approach [30]. Chen and Foloppe introduced a set of 30 carefully curated macrocycles and reported the performance of different MD-based approaches, the best two of which were the low mode MD approaches implemented within MOE [10] and Schrödinger’s MacroModel. Roughly speaking the more thorough ForceGen mode paralleled MacroModel’s performance, and the standard approach paralleled MOE’s performance, in both cases with ForceGen generating smaller conformer pools. More detailed performance comparisons will be made later, using larger data sets and more recent benchmarking results.

The BRIKARD approach was introduced as a sharp departure from stochastic sampling methods, instead employing methods from computational geometry to explicitly sample macrocycle ring conformations while maintaining closure constraints [30]. The method focuses on ring sampling, so the results for overall RMSD are perhaps not surprising (see top plot, Fig. 6), where the method performs substantially worse than either ForceGen mode (p-values $\le 10^{-5}$ by paired t-test). BRIKARD’s RMSD values were calculated using the automorph-corrected procedure used for all ForceGen results, with the “best-matching structures” provided in the BRIKARD report’s Supplemental Information [30].

Running *without* structure minimization, the BRIKARD method can be extremely fast using multiple computing cores (average time on the Chen/Foloppe Set of 81 s, compared with 132 s for ForceGen *-pgeom*). However, ring RMSD results (bottom plot, yellow line, Fig. 6) are significantly affected by the lack of minimization, performing much worse than *both* ForceGen modes or the standard BRIKARD procedure that includes minimization (p-values of 0.002 vs. ForceGen *-pgeom* and $\le 10^{-5}$ vs. ForceGen *-pquant* and standard BRIKARD). The standard BRIKARD approach (with minimization) running on a 40-core workstation, requires 2–3 times as long as ForceGen’s standard *-pgeom* mode on a 36-core workstation. ForceGen running on a single computing core is 10–15 fold faster than the BRIKARD approach. Note, also, that the ForceGen approach performs complete conformational search, rather than BRIKARD’s essentially exclusive focus on rings. ForceGen produces compact conformer pools (up to 250 or 1000 conformers), compared with the 10,000 conformers of the BRIKARD approach for the results depicted in Fig. 6.

Figure 7 shows typical examples of ForceGen performance in standard search mode, with best-matching conformer RMSD values and conformer search times indicated. We define the “total flexibility” of each macrocycle to be the sum of the freely rotatable bonds plus the number of single bonds within macrocyclic rings that are not primary amides. Four of the examples shown with total flexibility from 14–21 represent examples of targeted medicinal chemistry efforts. Of the 182 macrocycles, 127 had total flexibility of 21 or less, and using the standard search mode, the average RMSD was 0.61 Å, $\hbox{RMSD}^{95}$ was 1.5 Å, and the average search time was 33 s; performance was very close to that seen in the Platinum Diverse Set using the same *-pgeom* search mode. With macrocyclic search in the thorough *-pquant* mode, performance essentially matched that seen on the Platinum Diverse Set using the *-pgeom* mode. Macrocycles with total flexibility up to 21 appear to be completely tractable using the ForceGen approach. This size range covers medicinally interesting designed synthetic macrocycles, including HCV NS3-4A protease and BACE inhibitors [31–35].

The more flexible macrocycles within the ForceGen set (four examples are shown in Fig. 7 with flexibility of 25–29) tend to be exclusively natural products or direct analogs thereof. For this group, in standard search mode, the average RMSD was 1.2 Å, and in thorough search mode, the average RMSD improved only slightly to 1.1 Å. This level of performance is likely still sufficient for successful pose prediction and ligand design exercises, but the more flexible macrocycles are clearly more challenging. The plots in Fig. 8 illustrate the approximately linear relationship for ForceGen between ligand total flexibility and either accuracy or wall-clock time. For the standard search mode, timings were roughly 1 min or less, and thorough search required roughly twice as long, with no search times exceeding five minutes.

**Fig. 9:**
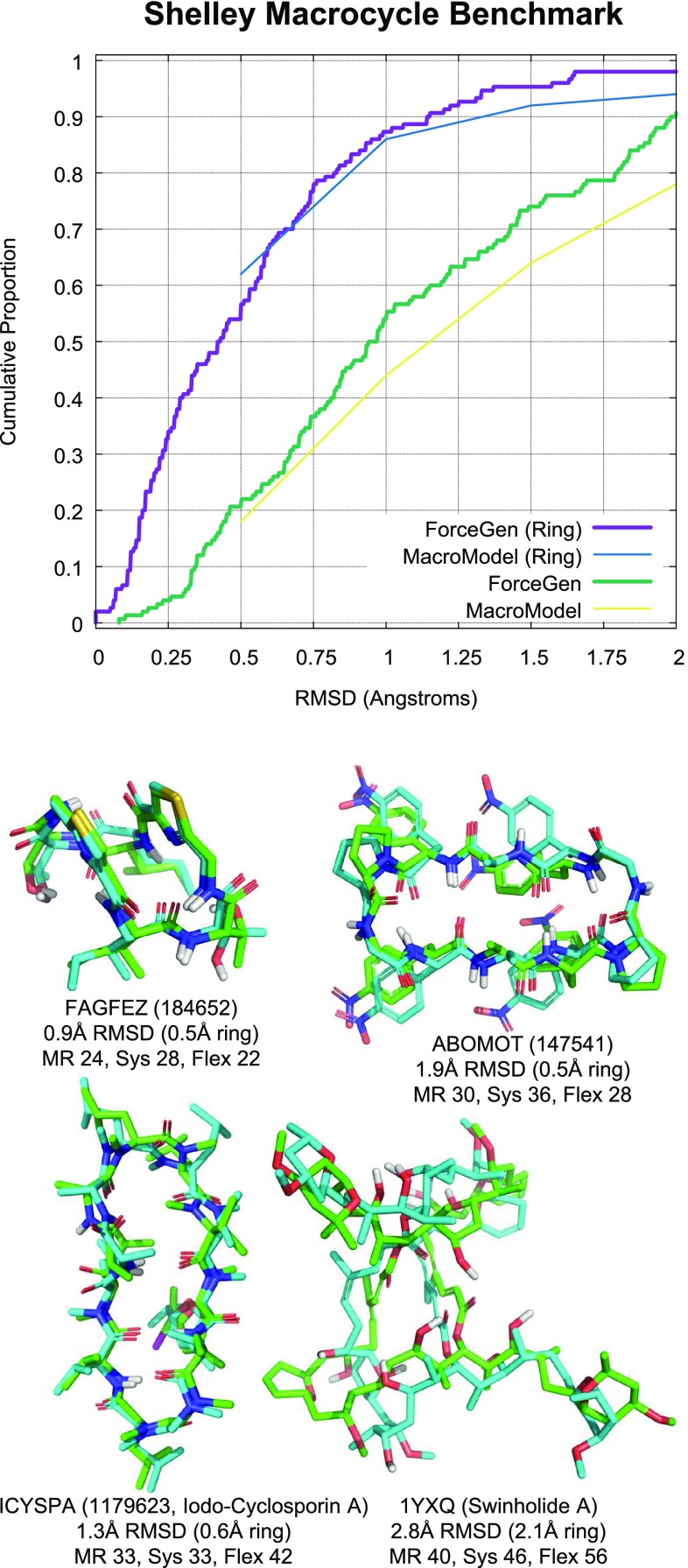
Performance of ForceGen (*-pquant*) on ring RMSD (thick purple line) and all heavy atom RMSD (green line) compared with results from the MacroModel approach (thin blue and yellow lines, test set values from [11]) using the 150 molecule Shelley Set. Three typical examples are shown (FAGFEZ, ABOMOT, and ICYSPA), each exhibiting stabilization with multiple internal hydrogen bonds. The last atypical example (Swinholide A) is a very large macrocycle with no specific stabilizing interactions

Direct timing comparisons with widely used methods will be made in more detail in the context of the Prime MCS Set, but it is important to note that macrocycle search times measured in a few minutes per molecule have been largely unheard of until very recently. Previous studies of macrocycle conformer generation have primarily made use of stochastic molecular dynamics methods [10, 11, 20, 36], with per-molecule search times typically measured in terms of multiple *hours* per ligand in order to achieve reasonable accuracy (MOE’s Low Mode MD and MacroModel’s Large-Scale Low-Mode search required 11,000 and 22,000 s on average for the Chen/Foloppe Set’s macrocycles [30]).

### Shelley macrocycle set: peptidic ligands

The Shelley macrocycle set was specifically curated to tune and test the MacroModel approaches for macrocyclic conformational search [11]. It contains 150 macrocycles, with a large fraction of larger peptidic rings that exhibit cross-macrocycle hydrogen-bonding in their experimentally determined conformations. Figure 9 shows a comparison between ForceGen’s thorough search mode and the test results from Shelley et al. All heavy-atom RMSD is shown (thick green line for ForceGen and yellow for MacroModel) as well as ring-atom RMSD (thick purple line for ForceGen and blue for MacroModel). For all-atom RMSD, ForceGen exhibited a clear advantage, with success rates of 10 points or more higher than those seen with MacroModel from roughly the 1.0 Å success threshold and up. For backbone ring RMSD, a smaller improvement was seen.

Figure 9 also shows four examples of ForceGen performance. The effects of stabilizing hydrogen-bonds in all but the case of Swinholide A, were a major feature of the Shelley Set that was clearly an under-represented phenomenon in the ForceGen Set. This was due to a difference in the curation of polymeric and non-polymeric ligands in the RCSB PDB, with the former not being represented in the “Ligand Expo”. Overall, the improvement in all-atom RMSD (*-pquant*) for the new ForceGen approach was 0.5 Å RMSD on average, and for the examples shown in Figure 9, the improvement was fully 2.0 Å RMSD.

The improvements from ForceGen version 4.0 to 4.4 on the Shelley Set were highly statistically significant (paired t-test p-values less than $10^{-10}$ for both the *-pquant* and *-pgeom* modes) for both conventional RMSD and ring-backbone RMSD. However, population-level success fraction differences were not large enough to make a firm conclusion about ForceGen compared with the MacroModel large-scale low-mode approach, though that will be possible using the Prime MCS Set in what follows.

Two points are important to raise in making the comparisons presented thus far. First, Shelley et al. [11] needed to manually correct over 50% of the the initial randomized ligand structures due to failures in SMILES to 3D structure generation (most of the failures were chirality inversions or incorrect configurations around double-bonds). Initial randomized ring RMSD values were approximately 1.1 Å. The approach taken with ForceGen validation was to use completely memory-free starting points, made by zeroing all atomic coordinates after noting chiral configurations and generating an initial 3D structure. This was an automatic (and failure-free) process that produced initial ring RMSD values averaging 1.4 Å.

The issue of memory-free starting points is also present in results reported for the BRIKARD method [30]. There, input seed structures were generated through dihedral perturbation and energy minimization *beginning from the experimental coordinates* and producing two seed structures with RMSD of at least 2.5 Å. To place this threshold value in context, nearly half of the Shelley Set’s ForceGen randomized structures had RMSD values between 3.1–7.5 Å. In order to avoid inadvertent bias, it is important for tests of conformational sampling adequacy to be started from coordinates with *no memory* of the correct configuration. This can be done either using SMILES as input to the procedure or by using zeroed 3D coordinates after making note of chiral atomic or bond configurations.

The second point is that the results reported by Shelley et al. [11] and Coutsias et al. [30] focused largely on ring RMSD rather than overall RMSD, following a somewhat common practice with macrocycles. Specific focus on ring-restricted RMSD may not provide a clear picture of real-world macrocycle sampling performance, for three reasons. First, given a macrocycle with little in the way of exocyclic components, the ring RMSD will track the conventional RMSD closely, so the former offers little extra information. Second, given a macrocycle with substantial exocyclic components, deviations from ideal ring geometries will generally be *amplified* in the conventional RMSD, making the ring-specific RMSD an overly optimistic measure of quality. Third, for any downstream usage of a macrocyclic conformational ensemble where the bioactive conformations are important, it will be the conventional RMSD that is most directly relevant to performance. Accurate ring RMSD with misplaced side chain conformations will generally not be relevant. The BRIKARD approach is entirely focused on ring geometry exploration; as such, it does not address the complete macrocyclic conformational sampling problem, and it will not be discussed further here.

### Prime MCS macrocycles

Recently, Schrödinger introduced a new method for macrocycle search [21], simultaneously introducing a new benchmark data set. The set was comprised of 130 CSD macrocycles, typified by having *minimal* exocyclic substituents, 60 PDB macrocycles that were a subset of the Shelley Set, and 18 examples from the PDB’s Biologically Interesting Molecule Reference Dictionary.

**Fig. 10:**
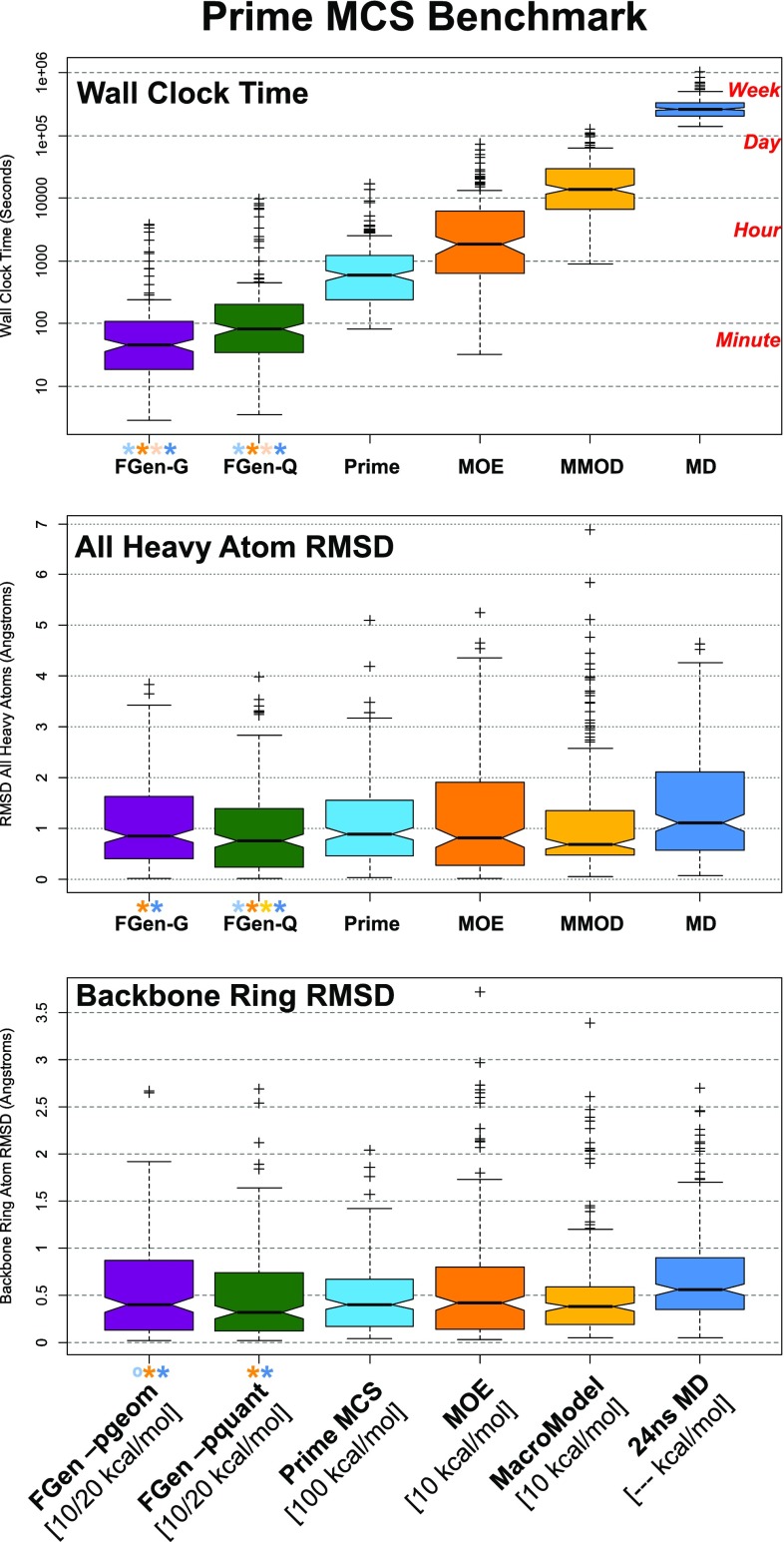
Performance of ForceGen compared with results from other methods on the Prime MCS Set. The notched box plots indicated the following: median (thick line), 95% confidence interval of the median (gap size of the box notch), 25th–75th percentile (inter-quartile range, or IQR, bottom and top of box), estimated non-outlier range (top and bottom whiskers, estimated based on IQR), and possible outliers (small plus signs). Paired t-tests were done between the ForceGen results and those of the other methods, with colored asterisks indicating superiority to the method with the indicated color and an open circle in the single case where any other method showed statistically better results than ForceGen (with p-values $< 0.05$)

**Fig. 11:**
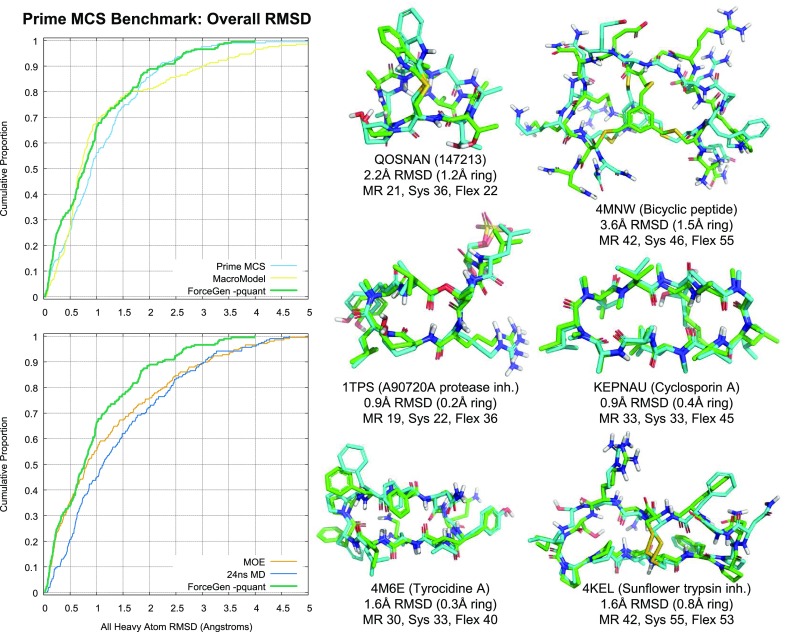
Detailed performance of ForceGen (-pquant) all atom RMSD compared with results from other methods on the Prime MCS Set. Compared with all four other methods, ForceGen’s performance improvement was statistically significant (paired t-test, p less than $10^{-4}$ vs. Prime-MCS and MacroModel, $10^{-7}$ vs. MOE, and $10^{-15}$ vs. MD)

Figure 10 shows box plots for wall-clock time, conventional RMSD, and ring RMSD for the ForceGen (standard and thorough modes), Prime MCS, MOE Low-Mode MD, MacroModel Large-Scale Low-Mode MD, and for a direct 24ns MD simulation run with Desmond [21]. By far, ForceGen was the fastest method, with wall-clock times generally in the seconds to minutes time-frame (for both search modes). Median times were 45 for the standard *-pgeom* mode and 82 s for the thorough *-pquant* mode. Average calculation times were 180 and 388 s, respectively (note that average times for all methods were skewed by a dozen or so outlier cases). Prime MCS was the next fastest, with respective median and mean times of 589 and 1067 s. MOE, MacroModel, and MD were the slowest, respectively with the following median/mean times (in seconds): 1,868/5,575, 14,004/21,671, and 263,960/291,924.

With respect to the timing results of the other methods, with the exception of the MD approach, the others were generated in a manner intended to produce reasonable ballpark estimates [21]. In the case of MD, a GPU-based calculation would have produced faster times, though certainly not enough to become competitive with the fastest methods. Prime MCS has the potential for speed increases by exploring different root macrocycle splits in parallel, and by merging the results of independent calculations [21]. However, it is not clear that an automatic procedure has been implemented, and the approach is limited by the effect on accuracy of merging the conformers produced by shallow explorations of multiple roots.

With respect to accuracy, the ForceGen *-pquant* approach was significantly better than all other methods in terms of conventional RMSD (by paired t-test, p $< 10^{-4}$ vs. Prime-MCS and MacroModel, $10^{-7}$ vs. MOE, and $10^{-15}$ vs. MD). It was also more accurate than both MOE and straight MD in terms of ring RMSD (paired t-test, p less than $10^{-4}$ vs. MOE, and $10^{-10}$ vs. MD). In no case was any other method superior by either conventional or ring RMSD than the ForceGen *-pquant* method, which produces up to 1000-member conformer ensembles.

For conventional RMSD, the *-pgeom* variant (which produces up to 250 conformers per ensemble) performed marginally better than (but statistically indistinguishably from) Prime and MacroModel and better than MOE and MD (paired t-test, p less than $10^{-2}$ vs. MOE, and $10^{-7}$ vs. MD). For ring RMSD, the *-pgeom* variant performed better than MOE and MD (p $< 0.05$ and p $< 10^{-5}$, respectively), and statistically similarly to MacroModel. Prime-MCS showed slightly better performance on ring RMSD than the ForceGen *-pgeom* variant (p = 0.001, by paired t-test).

Figure 11 shows full cumulative histograms of conventional RMSD for ForceGen’s thorough mode (thick green line) and the other methods (thin lines) along with examples of ForceGen results on six challenging examples from the Prime MCS Set. The QOSNAN example was the single case of all 208 where ForceGen performed poorly where all remaining methods performed reasonably well (for QOSNAN, the other methods yielded an average RMSD of 0.95 Å). It appears that some refinement of the bridge flipping procedure may be required, as both the bridge and both sides of the macrocycle are small and relatively rigid. In the five remaining cases, none of the other methods produced a result having RMSD within 2.0 Å. For 4MNW, the ForceGen result (3.6 Å) was not adequate for detailed modeling, but it was quite a bit better than the best of the other methods (which came from, surprisingly, the baseline 24ns MD trajectory, at 4.6 Å RMSD).

The 4MNW case was very unusual in one respect: the global minimum discovered was 60 kcal/mol *lower* than the local minimum energy (LME) obtained by minimization of the experimental 4MNW structure. This was 30 kcal/mol more extreme in such a deviation than the next most extreme case. The presence of mediating water molecules appears to influence the structure, but their presence should not create such a strong upward shift in the LME. It is the single case within the full Prime MCS data set where no method (including ForceGen) produced a conventional RMSD result better than 3.0 Å. Brief examination of the unbiased density around the modeled configuration of the macrocyclic ligand in 4MNW suggests that there is significant contiguous electron density present that was not modeled. This density may be partial density for another species, an alternative conformation of the existing macrocycle, or perhaps some other artifact. Symmetry related protein molecules also pack against the peptide binding site and could influence the observed conformer.

Energy windows for conformer generation on macrocyclic structures have been shown to have an effect on performance, with typical values being 10 or 20 kcal/mol [4, 10, 11, 20]. The ForceGen approach focuses search to identify conformers within 10 kcal/mol of the identified minimum, and, for macrocycles, adds novel conformers (discovered during intermediate search steps) up to a 20 kcal/mol window at the end of the search process. In the Prime-MCS study, both MOE and MacroModel were run with 10 kcal/mol windows. The straight MD simulation was run as a baseline control with no imposed energy window, simply recording 1000 snapshots (one every 24ps) without energy minimization or redundancy elimination. The Prime-MCS method imposed an energy window of 100 kcal/mol, five times larger than what has been typical in macrocycle search. It is not clear how this affected the relative energies of the closest matching conformers compared with the minima for the Prime-MCS results.

For ForceGen *-pquant*, the average energy above the discovered minimum for the best-matching conformer to the experimental structure was 7.3 kcal/mol. Roughly two-thirds of the best matches fell within a 10 kcal/mol window, with the remaining being 10–20 kcal/mol above the minimum.

Failures in sampling certainly exist when the global range of energies among a conformer pool produced from a memory-free starting point do not include the LME value obtained by minimization of the experimental structure. For ForceGen, there were seven cases where conventional RMSD was greater than 3.0 Å (defined as “failure cases”). The 4MNW case has already been discussed, and in four of the remaining six cases, ForceGen yielded conformer pools that covered the LME. This suggests that additional sampling using the current strategy might uncover closer conformations to the experimental one.

Two cases (1MIK and 2VYP, not shown) yielded global minima *higher* (by 5 and 8 kcal/mol respectively) than the LME, which may represent the absence of an important physical movement that would be required to uncover close-to-experimental conformations. These two cases represent failures for all methods tested, with an average of conventional RMSD values for the other methods being 3.6 Å and 4.6 Å, respectively, compared with ForceGen’s 3.3 Å and 4.0 Å. The ligand of 1MIK is a cyclosporin variant, but in contrast to the previous examples (see Figs. 9 and 11), here the molecule is bound to a protein and is nearly entirely hydrated. Rather than exhibiting the characteristic pattern of trans-annular hydrogen bonds present in the CSD structures, the structure everts and forms a closely packed set of hydrophobic interactions across the macrocycle. This conformation is, according to MMFF94sf, lower in energy than the best conformer found (which contains trans-annular hydrogen bonds). However, it requires coordinated movement of the backbone and the side chains in order to reveal it. For the ligand of 2VYP (the myxobacterial rhizopidin [37]), the bound conformation is also characterized by hydrophobic packing. However, in this case, much of the problem is simply its overall complexity: it has 30 rotatable bonds within the macrocycle and 36 outside of the macrocycle (the single highest total flexibility of all macrocycles studied here).

It is also possible to detect potential search strategy failures by identifying cases where a very small number of non-redundant conformations are produced on a very flexible molecule. For ForceGen, in its seven failure cases, this was not an issue, as it produced a minimum of 953 conformers for the set. MOE, on its set of 23 failure cases, produced an average of just 15 conformers each, with a maximum of 104, indicating a probable failure of search mechanics. MacroModel, on its 22 failure cases, produced an average of 386 conformers, roughly half of that which MacroModel produced for non-failure cases (which included conformer pools as large as 5705), suggesting some difficulty in sampling. Prime-MCS, on its seven failure cases, seemed to have a search-mechanics limitation on two cases, producing just 7 conformers for 4MNW and 290 for 4KEL, both being extremely flexible macrocycles.

The bottom four examples (1TPS, KEPNAU, 4M6E, and 4KEL) shown in Fig. 11 were challenging for all other methods. ForceGen’s conventional RMSD results were better than the average for the other methods by an average of 2.0 Å, and ForceGen’s results were better than the *best* of the other methods by an average of 1.2 Å. These four cases are clear examples where explicit sampling of hydrogen-bonding networks can be fruitful. Recall that the 4KEL example was used earlier (see Fig. 2) to describe aspects of the new ForceGen search procedure.

### Macrocyclic performance summary

The data sets explored here include 431 distinct macrocyclic ligand structures, spanning macrocycle ring sizes of 9–50 atoms, and including significant exocyclic flexibility in many cases. Across each different benchmark, the ForceGen thorough (*-pquant*) variant performed as well as or statistically significantly better than any other method, particularly with respect to conventional RMSD. The standard (*-pgeom*) variant performed equivalently to the best of the other methods in terms of conventional RMSD, while producing low-energy conformer pools of just 250 variants per molecule. The results involving macrocyclic structures with stabilizing trans-annular hydrogen bonding networks suggest a systematic advantage for such cases, but more extensive profiling of such peptidic macrocycles would be required to quantify any advantages there.

**Fig. 12:**
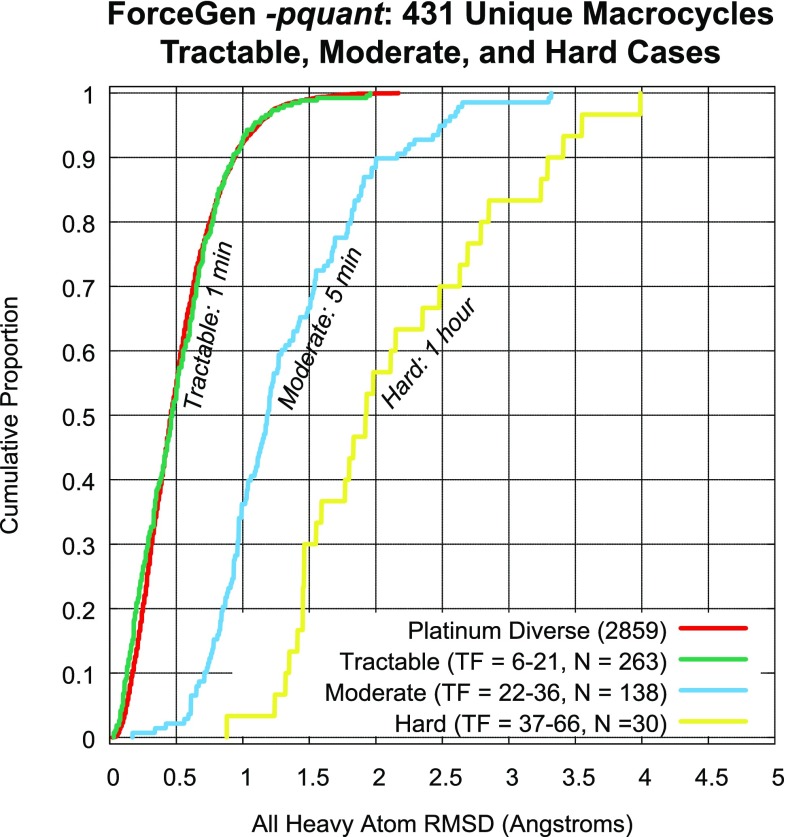
Over the 431 unique macrocycles studied, there were three clear classes of difficulty, broken down by total flexibility, with the tractable group yielding an RMSD distribution equivalent to that seen with non-macrocycles

While the performance gains exhibited by ForceGen are highly statistically significant in many cases, the *practical* significance of the accuracy improvements is difficult to assess. Macrocycles are quite a different area of study from the perspective of lead discovery and optimization than more standard non-macrocyclic ligands. For non-macrocycles, lead discovery through virtual screening is common and lead optimization assisted by consideration of large numbers of synthetically accessible analogs is standard. Computations that are directly dependent on conformational ensemble accuracy involving docking, ligand similarity, and binding affinity prediction are widely utilized for non-macrocycles.

In the case of a macrocyclic ligand, feasible synthetic variants are generally much fewer. Also, the calculations involving such molecules are more likely to involve the consideration of conformational ensembles, for example to estimate the likelihood of membrane permeability [38]. For macrocycles, it is not unusual to require deep analysis of a single molecule or just a handful of variants. In such cases, the *minimum* time required to produce biologically relevant conformational ensembles may be a serious bottleneck. ForceGen, even on a single computing core, is much faster than molecular dynamics approaches, including the low mode methods [10, 11, 20]. Through the multi-core implementation, ForceGen is much faster than all of the remotely competitive methods of which we are aware.

Figure 12 shows the conventional RMSD results for all 431 examples and places them within the context of performance on the Platinum Diverse Set as well as the complexity of the macrocycles. For 61% of the cases, with total flexibility of 6–21 (green curve), the RMSD distribution paralleled that seen with the large non-macrocyclic benchmark (red curve) almost exactly. Conformers were returned within 1.5 Å about 99% of the time, typically in about 1 min of wall-clock time. It is important to note here that these results are calculated with conventional RMSD, exactly the same for macrocycles and non-macrocycles. Ring RMSD is not relevant for contemplating conformational ensemble accuracy in normal predictive modeling workflows. This tractable size range includes designed synthetic macrocycles such as the HCV NS3-4A protease and BACE inhibitors seen in Fig. 7 [31–35]. For this class, interactive-time modeling of macrocycles for design appears to be possible, with results being suitable for downstream workflows that require accurate conformational ensembles.

The moderate complexity class (total flexibility 22–36) comprises 32% of the set. For these, search times were approximately 5 min, yielding accurate results ($\le$ 1.5 Å RMSD) about 70% of the time. In such cases, with the presence of some biophysical data to confirm ForceGen’s accuracy on a family of macrocyclic compounds, it should be possible in many cases to make use of the approach in detailed modeling studies.

The hard group of cases (total flexibility 37–66) represents just 7% of the overall set, but it includes interesting natural products that are biologically relevant. In this size range (roughly a cyclic decapeptide or larger), there are two challenges. In cases where there are clear topologically consistent hydrogen-bonding networks, ForceGen can often produce accurate results. However, the calculation time can increase to an hour or two on a modest 36-core workstation. Additional parallelization is possible, potentially using multiple multi-core computing nodes, to ameliorate this issue.

**Fig. 13:**
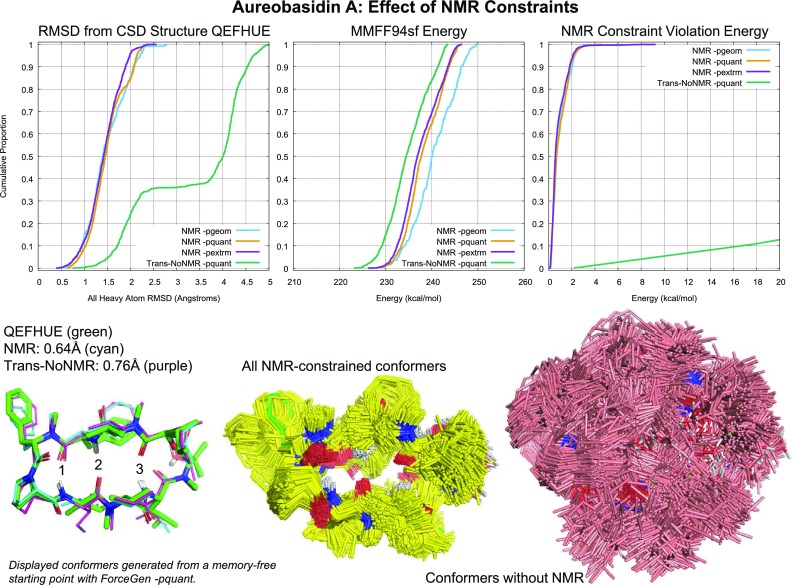
Ensembles generated with and without NMR distance and torsional constraints contain close-matching conformers to the CSD crystal structure QEFHUE (cyan and purple, left), but the constraints focus the conformational ensemble generated by ForceGen into a single coherent cluster (yellow, middle) rather than a mixture of different backbone motifs (salmon, right)

The bigger challenge is that, for very large macrocycles, ForceGen (or any other existing method) may not produce low-energy conformational ensembles that are sufficiently accurate to support further calculations and decision-making. In such cases, information derived from experimental biophysical measurements such as NMR may be exploited.

**Fig. 14:**
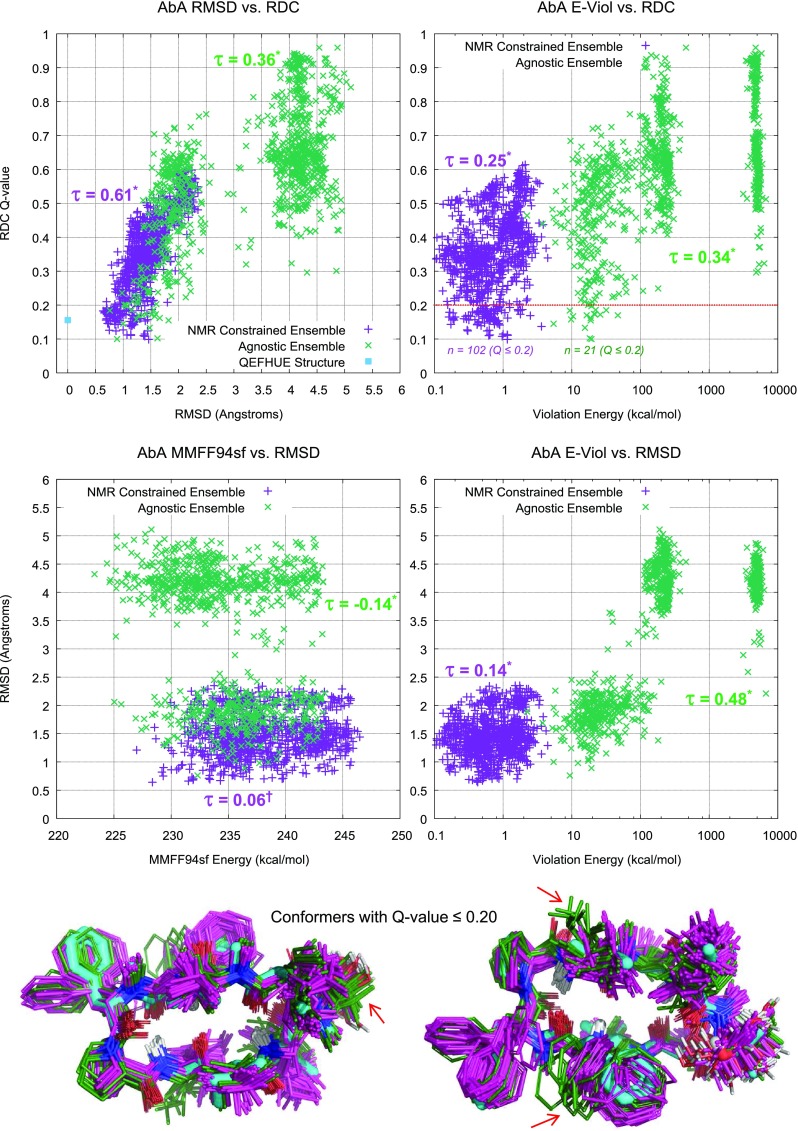
NMR-constrained conformer search (purple) and unconstrained search (green) produced substantially different ensembles, when considering the relationship between RDC Q-values and either RMSD or nominal NMR violation energies (top two plots) and also when considering the relationship between energetic components and RMSD (bottom plots); structural overlays (bottom) are for all conformers with Q-values $\le 0.20$, with carbon colors matching the plots

## NMR case study: Aureobasidin A

Aureobasidin A (AbA) is a depsipeptide antifungal, with *in vitro* activity against many pathogenic fungi [39]. It has 27 atoms within its macrocyclic ring, with 8 amide linkages, one ester linkage, and it includes a single proline residue. Four of the ring-nitrogen atoms are methylated, leaving three hydrogen-bond donor protons available for trans-annular interactions. ForceGen’s topological scan of possible hydrogen-bond triplets identifies a single possible trio, which are observed in the CSD crystal structure QEFHUE. This trio is evident in the depiction shown in Fig. 13, with the crystallographic structure shown in green, and the hydrogen bonding network appears to be stabilizing the backbone ring conformation (labeled 1–3).

NMR data were collected as described earlier, yielding 20 distance constraints and 3 torsional constraints in addition to the trans-proline constraint. Conformer pools for AbA were produced using the normal ForceGen modes (*-pgeom* and *-pquant*) along with a more thorough and time-consuming mode (*-pextrm*), both with and without the use of NMR constraints.

Beginning from a memory-free starting point, with *no* constraints of any kind, the ForceGen (*-pquant*) method yielded an ensemble with both trans- and cis-proline isomers, having a conformer within 1.2 Å RMSD of the CSD structure QEFHUE [24]. In the case of AbA, the *trans* proline isomer was of specific interest, though the cis isomer exists, with the two forms slowly exchanging [23]. The experimental NMR characterization was on the trans isomer (see Methods for details). Given that the NMR data characterized only the trans isomer, the computational experiments were done to parallel that choice. By adding a torsional constraint for trans-proline during the ForceGen conformational search, a slightly closer conformation to the CSD structure was obtained (0.76 Å, as seen in Fig. 13 in purple). In what follows, “agnostic” or “unconstrained” ForceGen calculations all refer to results produced using a single torsional constraint to enforce a trans-proline configuration. Without this single torsional constraint, the results do not change qualitatively, though the presence of a cis-proline population increases the proportion of the conformational ensembles in disagreement with trans-only NMR results.

Using the NMR data, the best match to the CSD structure was slightly closer (0.64 Å). However, a more significant effect was that the *entire* pool of AbA conformers shifted toward the QEFHUE structure. In principle, this need not have been the case, as the solution-behavior of AbA in the NMR solvent (DMSO) need not match the crystal form grown in ether [24] (just as the conformational preference for cyclosporin changes dramatically from solid-state small-molecule crystal structures to a hydrated protein-bound form in 1MIK). Essentially all of the conformers discovered using the three ForceGen search modes under NMR constraints were within 2.0 Å RMSD, with the entire (*-pquant*) pool shown in yellow in Fig. 13 (middle bottom). By contrast, about one-quarter of the conformations elucidated without NMR constraints were close to the crystal structure (upper left plot, green curve).

ForceGen seeks to optimize a combined energy function, which is the sum of the MMFF94sf energy plus the violation energy from the NMR-based constraints. Recall that the latter are expressed as square-welled quadratic functions centered on the experimentally observed expected distance or torsional value. Figure 13 shows the distributions of the two components of the energy terms. In the agnostic conformer pool, the distribution of MMFF94sf energy was centered on a median of 236 kcal/mol (green curve, middle plot), with the most thorough search under NMR constraint being shifted roughly 3 kcal/mol higher (purple curve, middle plot). The small gap between the no-NMR and NMR cases reflects some tension between attempting to satisfy the experimental constraints while still respecting the energetics of the force field. Because experimental restraints are incorporated as a penalty function, the distribution shifts towards slightly higher energies, as expected.

The distribution of violation energy values is more interesting (right plot). Each of the ForceGen search modes under NMR constraint produced ensembles with roughly 90% of the conformers having less than 2 kcal/mol in terms of energetic penalty violations. By contrast, without using the constraints, just a few percent of the ensemble had less than 5 kcal/mol violation energy when profiled *post facto* using the NMR data. If the force field were an extremely good predictor of AbA behavior in solution (and was very effective in search), then one would expect the ensembles to be relatively similar. However, we see instead that the NMR information had a large influence on the outcome of the search, both as measured by closeness to the CSD structure and congruence with the NMR data.

To further understand the extent to which the conformer ensembles reflected relevant behavior of AbA, RDC data were used as an orthogonal validation (see NMR experimental details in the Methods section). For each conformer within the NMR-constrained and unconstrained *-pquant* pools, the correlation with the RDC data was calculated. Similarly, the RDC Q-factor was also calculated for the crystal structure QEFHUE (0.156). It is important to note that RDC correlation is a measure of a solution conformational ensemble; however, in this case, the QEFHUE crystal structure is representative of a conformation in solution, reflected by its low Q-factor. Figure 14 shows a plot of RMSD to the QEFHUE crystal structure (top left, X axis) and RDC correlation Q-values (Y axis) for the NMR-constrained pool (purple) and the unconstrained pool (green), with both pools arising from the ForceGen search protocol beginning from the same memory-free starting point. As would be expected, the NMR-constrained pool had much lower overall Q-values (0.37±0.11) than the unconstrained pool (0.60±0.17) (p-value by t-test $\ll 10^{-6}$). For both pools, the correlation was highly statistically significant (Kendall’s Tau values are shown, with the asterisks indicating p $< 10^{-5}$). The NMR-constrained pool formed a single population of variants. The agnostic pool formed two populations, with one overlapping the NMR-constrained pool, and the other forming a very different set of configurations (this is also seen in Fig. 13). Note that the conformational search identified a number of conformers with Q-values as good or better than the QEFHUE crystal structure (0.156); there were 5 such conformations in the agnostic ensemble and 31 in the NMR restrained ensemble.

The violation energy of the quadratic constraints from the NMR data is shown for the constrained pool relative to RDC Q-values (top right, purple for the NMR-constrained pool). All values were quite low, as would be expected given non-conflicting constraint data. The nominal violation energies were calculated for the agnostic pool, and they are also shown plotted against RDC Q-values (green). The population of low RDC Q-value conformers was much smaller (21 compared with 102 conformers for the NMR-constrained pool with Q $\le 0.20$) and the violation energies were much higher, forming three groups. The group with the smallest violations (roughly 10–20 kcal/mol in quadratic penalty values) had RDC Q-values from roughly 0.1–0.6 (essentially the same range as for the NMR-constrained pool). The next group had more severe distance violations (100–500 kcal/mol penalties), and none of these conformers had very low Q-values. The last group contained both distance and torsional violations, the latter of which, being penalized proportionately to squared degrees of deviation, produced very high nominal violation energies.

The effect of including the sparse NMR constraints was to eliminate all conformers above 2.5 Å RMSD or with Q-values above 0.6. While ForceGen was able to identify exemplars with the low RMSD, low Q-value, and relatively low NMR violations, it clearly identified a significant fraction of conformations that have no basis of experimental support. The highly statistically significant correlations between Q-values and both RMSD *and* quantitatively measured NMR violations over the range of 0.1–0.6 suggests that Q-values within this range may be relevant beyond the extremely low end, so it may be possible that some of the high-RMSD agnostic conformers that are distinct from the NMR-constrained pool have some relevance.

The bottom two plots of Fig. 14 show the relationship between the MMFF94sf energy and violation energy with RMSD. The latter (bottom right plot) shows a clear relationship, with low NMR violation energy clearly being correlated with low RMSD from the CSD crystal structure QEFHUE. However, there is only a *very* weak correlation between force field energy and RMSD within the NMR-constrained pool (Kendall’s Tau of 0.06, p $< 0.01$ indicated by a dagger in the Figure). The correlation is *negative* for the agnostic pool, owing to the population of high-RMSD conformers with lower energy than those having lower RMSD. This was as expected: pools of conformers within 20 kcal/mol of the identified global minimum by ForceGen very often contain biologically relevant conformers. But the average gap in energy between the lowest energy conformer and the best biological match was roughly 7 kcal/mol across the macrocycle benchmarks described above.

The bottom of Fig. 14 shows all conformers that had RDC values of 0.20 or better (front view at left as in Fig. 13 and flipped top to bottom at right, with carbon colors matching the plots). Both of the ForceGen pools covered the crystal structure with multiple variants, though additional side chain movements were evident (e.g. the phenylalanine at the top left of the left-hand overlay). In a few positions (indicated by red arrows), the low-Q-value agnostic pool (green) showed slightly more movement than the NMR-constrained pool (purple), but both pools covered roughly the same movements.

It is also possible to characterize fidelity to experimental NMR data by considering average inter-proton distances (e.g. as in [40]). For the NMR-constrained ensemble of Fig. 14, 18/20 of the inter-proton distances had mean differences from the experimental ideal of 0.5 Å or less, and the remaining two had mean deviations of 0.7 Å and 0.9 Å. Recall that the NMR constraints were introduced as a middle distance, with ± 0.5 Å allowance (see Methods under “NMR Constraints”), so these deviations of the inter-proton means indicated no violations on average for 18/20 of the imposed constraints and just 0.2 Å and 0.4 Å for the remaining two.

For the unconstrained pool, 5/20 had mean differences from the experimental ideal of 0.5 Å or less (thus falling within the contraint window). However, this mainly reflects the diversity of conformations within the unconstrained pool. There was a population (see Figs. 13 and 14) within the unconstrained pool that had minimal NMR violations, low RMSD to the QEFHUE crystal structure, and very good agreement with the RDC data. Average inter-proton distances within an ensemble will not, in general, match experimental expectation if there is both a “correct” population and an “incorrect” one within the ensemble.

Clearly, NMR constraints had a large impact on the degree of effective sampling of biologically relevant conformers. Using just 20 distance constraints and 3 torsional constraints from NMR data (plus the single trans-proline torsional constraint), the entire ForceGen conformational ensemble for aureobasidin A was focused within an experimentally relevant space, confirmed by dramatic downward shifts in the distributions of both RDC Q-values and RMSD from a small molecule crystal structure.

NMR-based analysis and design of peptidic macrocycles is an area of increasing interest, and three recent papers are notable in the context of the work presented here. Kamenik at al. [40] studied three small peptidic macrocycles (two with five residues, which fall well within our tractable range, and one with six which falls into our moderately tractable range). They considered the relationship between conformational ensembles generated by accelerated molecular dynamics compared with experimental NMR data. They showed very good agreement between aMD ensembles and NMR inter-proton distances, and it will be interesting to assess the ForceGen approach on peptidic macrocyles in this smaller size range in the context of NMR data.

Witek et al. [41] characterized the behavior of cyclosporin A in aqueous and apolar environments, both using molecular dynamics and NMR approaches. Using MD simulations of 100ns in length, they did not observe conversion from a closed cyclosporin seed conformation to an open one in water, nor did they observe a transition from an open seed conformation in chloroform to a closed conformation that agreed with crystallographic or NMR experimental data. Using 100 diverse seed conformations, a more complete characterization was possible, both in terms of conformational ensembles and kinetic behavior, with better agreement to experiment. Such challenges in sampling complex macrocycles using MD echoed the MD results discussed here in the context of the Prime-MCS set.

Baker’s group has addressed the prospective design of macrocycles with desired conformational behavior (i.e. the presence of stabilizing trans-annular and exocyclic hydrogen bonding networks) [42]. In 9 of 12 cases, computationally designed macrocycles exhibited NMR profiles where corresponding conformational ensembles matched the designed structures. This clearly is a direction in which future ForceGen work will be directed.

## Conclusions

The results reported here for ForceGen conformer generation are comprehensive. For non-macrocycles, conformer pools were generated for both screening and pose prediction applications, mirroring the benchmarking done for numerous other methods on the Platinum Diverse Set of 2859 PDB ligands [19]. ForceGen is faster than all other benchmarked methods while producing equally accurate results in terms of mean RMSD from experimental structures. Because the approach does not make use of templates or torsion libraries, it is able to maximize the diversity of conformer pools, subject only to energetic considerations. This results in significantly better coverage of conformational space, as measured by the maximal observed RMSD covering 95% of the overall set ($\hbox{RMSD}^{95}$). On a single computing core, for processing very large databases for virtual screening applications, ForceGen produces conformer pools (with a maximum size of 50) in a median time of 0.59 s (mean time of 1.35). It does so beginning from standard molecular file inputs (either 2D or 3D), with no special requirements for specification of templates or other information for either macrocycles or non-macrocycles.

Multiple parts of the ForceGen algorithm are parallelizable, and this provides significant speed increases that are particularly relevant in interactive-time modeling, for example in lead optimization using a docking workflow. Here, using a modest multi-core workstation, typical search times were 1–3 s per molecule for accurate search (*-pgeom* level, 250 conformer pools), which yielded a mean RMSD of 0.55 Å and $\hbox{RMSD}^{95}$ of 1.22 Å. For comparison, the most accurate results from other benchmarked methods were 0.57 and 0.58 Å (mean RMSD, OMEGA and ConfGenX, respectively) and 1.4 Å $\hbox{RMSD}^{95}$ for both OMEGA and ConfGenX. Times for OMEGA and ConfGenX were 2–3 and 13–14 s, respectively. ForceGen’s faster search for pose prediction (*-pgeomf*) produced mean RMSD of 0.58 Å and 1.27 Å $\hbox{RMSD}^{95}$ with mean and median times of 0.61 and 1.53 s using the multi-core calculation option.

For macrocycles, where the typical application scenario often includes a single molecule or a handful, the multi-core option yields larger performance gains. Of the 431 unique macrocycles studied here, 61% fell into a tractable class, defined as having 21 or fewer total rotatable bonds (both macrocyclic and exocyclic). Conventional RMSD performance on this set matched performance on the full Platinum Diverse Set of *non-macrocycles*, with mean RMSD of 0.51 Å, and with search requiring 58 s on average of wall-clock time (thorough search mode). This class of macrocycles includes many that have been the subject of synthetic drug design efforts. Interactive-time modeling of such molecules with accuracy sufficient to support design-oriented workflows offers new opportunities in exploiting synthetic macrocycles within this complexity class. Moderately tractable cases (total flexibility 22–36) formed about one third of the 431, with mean RMSD rising to 1.32 Å, and search times increasing to about 5 min. Effective docking strategies for linear peptides have been recently shown for peptides of 3–12 residues in times of 15–40 min [43], and the smaller range of cyclic peptides fall into the tractable class described here.

Just 7% of the macrocyclic examples fell into the “hard” group, where both accuracy decreased and computational cost increased. While the computational cost aspect is a manageable engineering problem, the accuracy issue is deeper. However, as seen with Aureobasidin A, ForceGen can utilize NMR constraints in a natural fashion, even relatively sparse constraints. The enhanced biological fidelity of the conformational ensembles for such complex macrocycles should be beneficial in developing predictive models for critical aspects of macrocycle behavior. Further, it is likely that the method can be used iteratively within the context of the NMR data analysis workflow to help in the identification of correctly assigned signal peaks, which would reduce the burden of traditional NMR data analysis.

The larger macrocycles are very often natural-product based and often contain peptide backbones. Cyclic peptides lacking proline residues form a particularly interesting computational challenge because the backbones are all just poly-glycine. Conformational preferences for such molecules are driven by N-methylation patterns (which modify the available hydrogen-bond networks), bridging features such as disulfides, and the side chains present at each position. ForceGen’s approach never breaks a macrocycle nor treats a macrocycle backbone as something that can be considered as separate from its pendant functionality. Strategies such as those employed by Prime-MCS and BRIKARD [21, 30] make strong assumptions about independence: Prime-MCS assumes that relevant dihedrals can be derived without an intact macrocyclic ring, and the BRIKARD approach assumes that side chains are irrelevant while generating ring configurations. ForceGen continuously explores the low energy landscape of a macrocycle using natural physical movements, never breaking a ring, and with all calculations done in the presence of all atoms.

ForceGen relies on modified version of MMFF94s, which was parameterized for small molecules, not peptides. In this work, peptidic macrocycles tended to be much larger than non-peptidic ones. However, within the smaller size-range of peptidic macrocycles, we did not observe any systematic differences in predictive accuracy between peptides and non-peptides of similar complexity. Clearly, there is room for improvement in terms of the underlying force field, particularly in the area of topologically distant non-bonded interactions. In principle, other force fields would be of interest to explored within the ForceGen framework, though substantial effort would be required in order to match the existing implementation’s speed.

Despite there being a significant and medicinally relevant portion of the macrocyclic space that appears to be tractable, the central macrocycle conformational search problem is not yet solved. There is room for improvement in both search algorithms as well as in the details of energetic scoring. It is likely that improvements in the sampling tools such as those reported here will help to generate more experimental data on macrocyclic behavior. To the extent that happens, especially regarding data in aqueous solution and membranes, it will be of great benefit to supporting further improvement of algorithms and force fields.
